# Thirty Years of Borophene: Origins, Scientific Progress, and Future Directions

**DOI:** 10.1021/acsami.5c19994

**Published:** 2026-01-22

**Authors:** Thiago F. Santos, Ihsan Boustani, Caroliny M. Santos, Dieter Rahmadiawan, Shih-Chen Shi, Jose Heriberto Oliveira Nascimento

**Affiliations:** † Postgraduate Program in Chemical Engineering, 28123 Federal University of Rio Grande do Norte, Natal, Rio Grande do Norte 59072-970, Brazil; ‡ GPIMN-Micro and Nanotechnologies Innovation Research Group, Federal University of Rio Grande do Norte, Natal, Rio Grande do Norte 59072-970, Brazil; § Theoretical and Computational Chemistry, Faculty of Mathematics and Natural Sciences, 26603 Bergische Universität Wuppertal, Gauss Strasse 20, Wuppertal D-42097, Germany; ∥ Department of Mechanical Engineering, 34912 National Cheng Kung University (NCKU), Tainan 70101, Taiwan; ⊥ Department of Mechanical Engineering, Universitas Negeri Padang, Padang, Sumatera Barat 25173, Indonesia

**Keywords:** boron, density functional theory, DFT, graphene, monolayer

## Abstract

Borophene, an atomically thin boron sheet, exhibits exceptional polymorphism, metallic conductivity, and anisotropic mechanical behavior. Interest in borophene is growing, reflected in the increase in scientific publications and international collaborations. The 2D materials market is projected to reach a size of 2030, and technology companies are investing in its commercialization, especially for flexible electronics and energy storage. This perspective integrates a comprehensive bibliometric analysis with a critical evaluation of theoretical predictions, experimental synthesis methods, phase stability, and ambient reactivity. In epitaxial growth, emerging wet-chemical and exfoliation routes and functionalization strategies. The influence of computational methodology on predicted properties is discussed, highlighting inconsistencies among PBE, hybrid, and correlated approaches. Finally, we outline key challenges, including air stability, scalable synthesis, and device integration, and propose research pathways needed to translate borophene from fundamental discovery to practical technologies.

## Graphical abstract

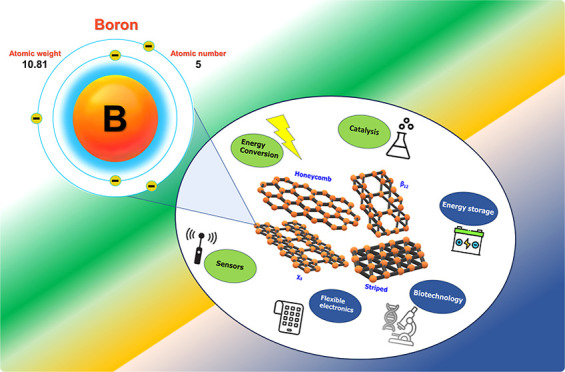

## 1 Introduction

### 1.1 Background on Boron and 2D Materials

Boron, a light element with atomic number 5, has unique chemical and physical properties due to its electron deficiency and ability to form diverse bonds.^1^ Unlike carbon, which tends to form stable three-dimensional structures like diamond and graphite, boron is known to create complex clusters and networks, resulting in a wide structural diversity.^2^ In recent decades, the discovery of two-dimensional (2D) materials, beginning with graphene, has revolutionized materials science by highlighting the potential of atomic sheets with exceptional electronic, mechanical, and chemical properties. Given boron’s long history of complex cluster chemistry, the emergence of 2D materials naturally led researchers to explore whether boron could form atomically thin sheets, thereby laying the conceptual foundation for borophene. The term boron clusters was coined by Lipscomb^3^ and Longuet-Higgins^4^ in the 1950s through the theoretical prediction of borane [B_12_H_12_] and through their pioneering work on the chemical bonds of borane, including the concept of the three-dimensional icosahedral clusters. Their work led to the first synthesis of stable dodecaborate [B_12_H_12_]^2–^ by Hawthorne^5^ and Pitochelli^6^ in the 1960s. Lipscomb, the Nobel Prize winner, studied, in addition, carboranes and other compounds like those containing boron–hydrogen bonds. The most important research was the focus on their three-dimensional structures and the rules of chemical bonding that govern them using sophisticated techniques like X-ray crystallography and quantum mechanical calculations, providing a foundation for understanding the complex chemistry of boron. In this context, boron emerges as a promising candidate for 2D materials since small boron clusters can adopt planar or nearly planar geometries due to multicentric bonding. Furthermore, extensive borophene sheets are chemically reactive because their electron-deficient structure exhibits subcoordinated surface sites. Thus, the geometric planarity of borophene does not contradict its chemical instability under ambient conditions. The study of 2D boron, or borophene, arises from the convergence of theoretical predictions and experimental advances, offering a platform for exploring novel physical phenomena and innovative technological applications.

### 1.2 Why Boron Sheets?

Boron sheets, or borophene, stand out among 2D materials for several reasons. First, boron’s electron-deficient nature allows for the formation of multiple polymorphs, allowing for the tunability of properties such as conductivity, anisotropy, and chemical reactivity.^7^ Second, borophene exhibits exceptional mechanical properties, including high strength and flexibility, superior to those of many other 2D materials. Furthermore, boron’s rich chemistry favors functionalization, intercalation, and hybridization, expanding its potential applications in areas such as energy storage, catalysis, sensors, and superconductivity.^8,9^ The “rich chemistry” of boron refers to its ability to form multicenter bonds, electron-deficient frameworks, and strong interactions with electronegative atoms such as O, N, F, and H.^10^ These features enable diverse functionalization pathways, including oxidation, fluorination, hydrogenation, and substrate-induced reconstruction, which allow tuning of borophene’s electronic and structural properties. Additionally, theoretical studies indicate that boron sheets can harbor exotic phenomena such as Dirac fermions, topological phases, and superconductivity, solidifying borophene as a unique platform for fundamental research. The combination of structural versatility, exceptional properties, and potential for new applications explains the scientific community’s growing interest in this material.

### 1.3 Scope of the Review

Several reviews have summarized specific aspects of borophene, including its synthesis and electronic properties.^11,12^ The present review extends these works by (i) incorporating a detailed bibliometric analysis of research trends, tracing its development over the past three decades, (ii) critically comparing synthetic reproducibility across substrates, (iii) consolidating recent progress in wet-chemical and solution-phase approaches, (iv) examining methodological divergences among PBE, hybrid, and correlated computational methods, and (v) presenting an updated perspective on air stability, scalability, and integration. This comprehensive overview of borophene addresses theoretical predictions and early experimental achievements, including structural diversity, synthesis methods, and characterization techniques for boron sheets. Unlike typical reviews that mainly present the known properties and applications of borophene, we highlight unexplored possibilities and new horizons for this material. The paper discusses in detail the gaps in knowledge related to the synthesis, theoretical models, and perspectives of borophene. It points out new areas for future research. In this review, the potential applications of boron-based 2D materials are discussed thoroughly, ranging from energy storage and catalysis to flexible electronics, with a focus on the new challenges of their environmental stability and large-scale production.

## 2 Methodology of Bibliometric Analysis

Figure 1 presents the data collected from a bibliometric analysis of the boron sheet and boron cluster, focusing on the evolution of the borophene field over 30 years (1987–2024). The methodology adopted was divided into two main stages: data retrieval and result interpretation.

**Figure 1:**
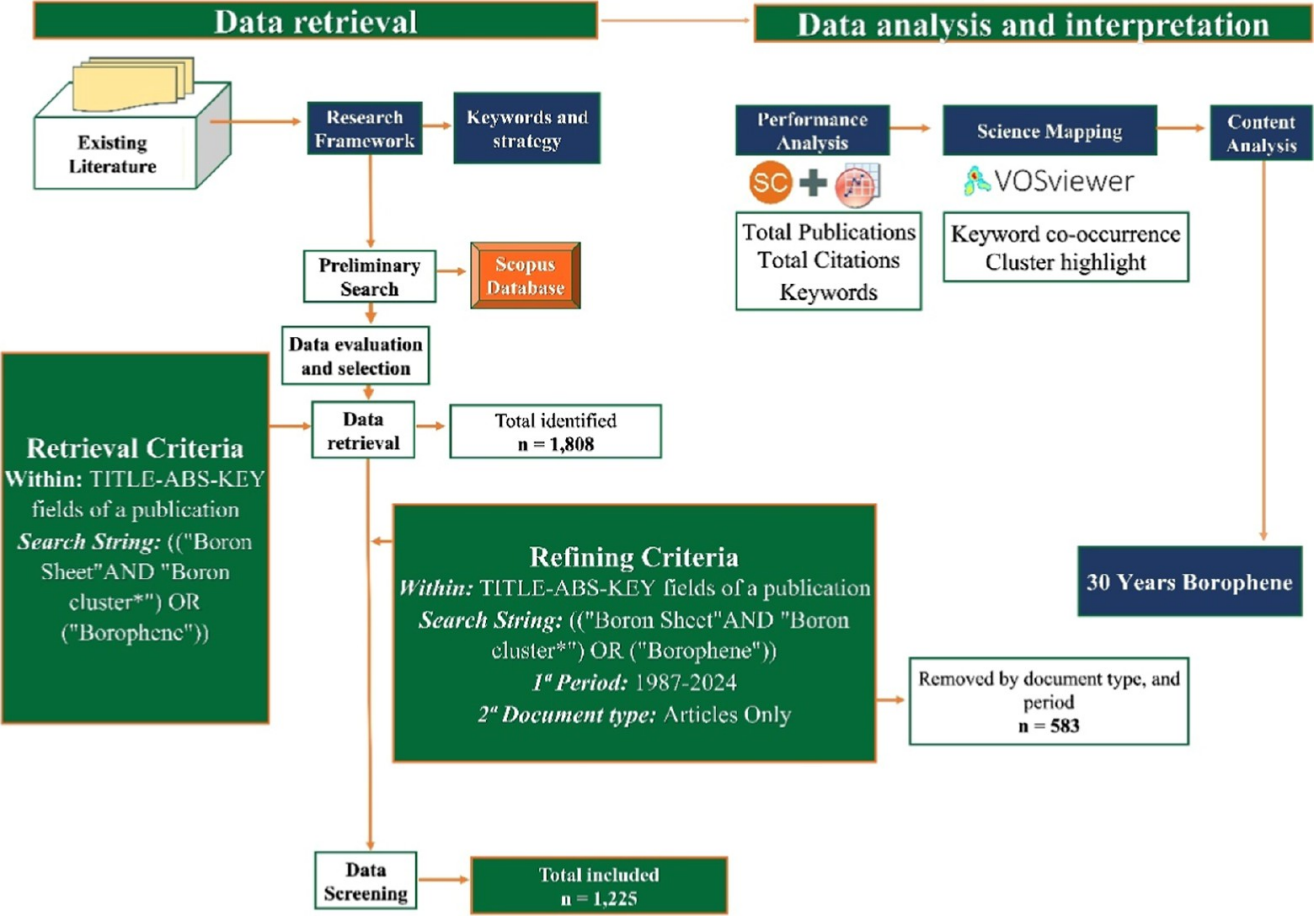
Toolbox used in bibliometric analysis.

### 2.1 Type of Study

This study is a quantitative and descriptive bibliometric analysis focused on the scientific literature related to boron sheets and boron clusters, with the objective of mapping the evolution of the borophene research field over a 30 year period (1987–2024).

### 2.2 Database

The data were collected from the Scopus database (Elsevier), chosen due to its broad multidisciplinary coverage and the reliability of its indexed scientific records.

### 2.3 Search Strategy

A structured search string was defined using the keywords: ((“boron sheet” and “boron cluster*”) OR (“borophene”)). The search was applied to the TITLE-ABS-KEY fields (title, abstract, and keywords). In the initial search, 1808 documents were identified. Refinement filters were then applied for time frame (1987 to 2024), and document type (only scientific articles). After applying these criteria, 1225 articles were selected for final analysis. The data collection was completed on November 28, 2025.

### 2.4 Inclusion and Exclusion Criteria

Inclusion Criteria: Peer-reviewed scientific articles, publications in English, and studies within the period 1987–2024. Exclusion Criteria: Reviews, conference papers, book chapters, and nonpeer-reviewed documents. Publications outside the thematic scope are defined by the keywords.

### 2.5 Data Extraction and Treatment

Bibliographic data were exported from Scopus in CSV format, including: title, authors, year of publication, institutional affiliation, country, journal, keywords, and number of citations. The data set was processed and organized in spreadsheets (Microsoft Excel) to support descriptive and statistical analyses and visualization (Origin).

### 2.6 Tools and Software Used

The bibliometric analysis was conducted using VOSviewer: for performance analysis and generation of coauthorship, cocitation, and keyword co-occurrence networks, as well as identification of thematic clusters. Microsoft Excel: for preliminary data processing and organization.

### 2.7 Bibliometric Indicators

The main indicators assessed included: Annual scientific production to identify temporal trends, citation performance, keyword co-occurrence, and thematic cluster identification, allowing the synthesis of trends, advances, and emerging themes in the borophene research field.

## 3 Scanning the Trajectory of Borophene

### 3.1 Historical Trends

Figure 2 presents three data points on the evolution of publications: citation impact and distribution by language (English, Chinese, German, and Persian) over the period 1987 to 2024. Figure 2a shows the annual number of published articles. Throughout the period, 180 scientific articles were published, showing growth, especially from the 2000s onward. Initially (1987–1996), only 3 articles were published by 1996. Starting in 1997, publications increased steadily until 2007. In 2008, 8 articles were published, and the output was accelerated. The publication CAGR from 2012 to 2018 was 57.62%; publication peaked with 11 articles in 2013, 15 in 2014, and 92 in 2018. This trend reflects rising global interest in two-dimensional materials like borophene. After 2018, the publication CAGR was 20.64%, and publication rates remained high and grew further, forming an almost exponential curve over 30 years. Citation analysis helps identify influential publications, emerging subfields, and shifts in research emphasis. For borophene, citation patterns reveal the dominance of early theoretical predictions, the central impact of the 2015–2016 experimental synthesis papers, and a growing emphasis on stability and device-integration studies. Thus, citation data provide a quantitative perspective on research momentum, community focus, and scientific influence. As shown in Figure 2b, the data show that from 1987 to 1992, citations were very low, with only 10 over six years. Starting in 1994, there was modest growth, with increases until the late 1990s. A significant surge occurred in 1998 with 12 citations, followed by more increases. In 2002 and 2003, citations rose to 47 and 65, indicating growing interest. From 2005, growth accelerated, reaching 116 in 2005, then 136 in 2006, and 139 in 2008. After 2010, citations entered the hundreds, peaking in 2016 with 691. The citation CAGR from 2012 to 2018 was 55.41%, indicating sustained expansion of the field. From 2017 to 2024, impact peaked with thousands of citations in 2017 and 2018 and 7379 in 2024. Overall, citations surpassed 37,000, reflecting boron’s rising influence. Figure 2c shows the contribution by language. Thus, 98.6% of the articles were published in English, while only 1.3% were in Chinese, and 0.1% were in German and Persian. These data reinforce the dominant role of English as the primary language of international scientific communication in the field of borophene.

**Figure 2:**
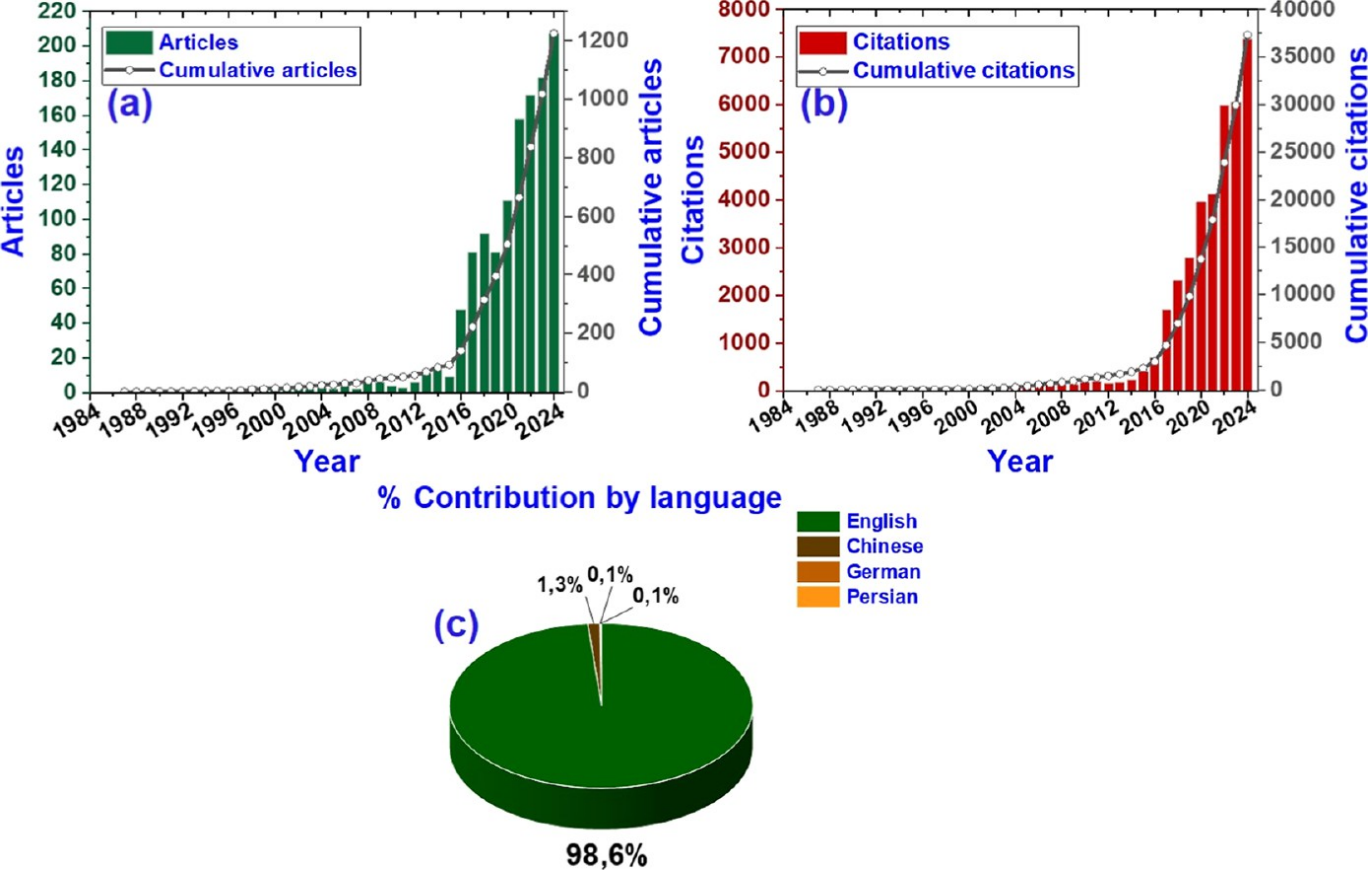
(a) Articles and cumulative, (b) citations and cumulative, and (c) percentage distribution by language from 1984 to 2024 obtained from the Scopus database.

The limited progress shown in Figure 2 reflects several scientific and technical bottlenecks. Borophene growth is strongly substrate-dependent, and only a few metal surfaces (Ag(111), Au(111)) stabilize the desired phases.^13^ Its rapid oxidation prevents extensive ex-situ characterization and device assembly.^14^ Reproducible phase control remains challenging due to competing polymorphs with small energy differences.^15^ Scalable routes beyond UHV techniques are still under development. Overcoming these constraints will require improved in situ growth monitoring, exploration of alternative substrates, encapsulation strategies such as h-BN lamination, and standardized ambient-stability assays.^16,17^ These steps are expected to accelerate borophene’s transition from fundamental study to practical applications.

### 3.2 Research Networks

Figure 3 presents two analyses focusing on contributions by country and by field or department in document production. As shown in Figure 3a, the data shows countries’ contributions to scientific publications. China (CTR 1) leads with over 50%, dominating borophene and 2d materials research. The US (CTR 2), India (CTR 3), Iran, Germany, Turkey, Japan, and Singapore are also major contributors, with the Russian Federation and Saudi Arabia in the top 10. This highlights the global and investment-driven nature of materials science research. As shown in Figure 3b, the text shows contribution percentages by department: Materials Science (DPT 1) leads with 26.18%, followed by Physics & Astronomy (DPT 2) and Chemistry (DPT 3). The study focuses on boron atomic structures, requiring analyses of chemical bonds, cluster stability, and electronic traits. Physics supports theoretical modeling of borophene’s properties, essential for its tech applications. Materials science explores uses in electronics, batteries, sensors, and more, emphasizing innovation. Engineering, energy, and chemical engineering contribute with research on electronic components, nanodevices, and energy storage, also aiding synthesis, scaling, and industrial viability. The other fields cited in Figure 3b, although with a smaller volume of publications, demonstrate the multidisciplinary nature and technological potential of borophene, connecting fundamental research with applied innovations in various fields of science and engineering.

**Figure 3:**
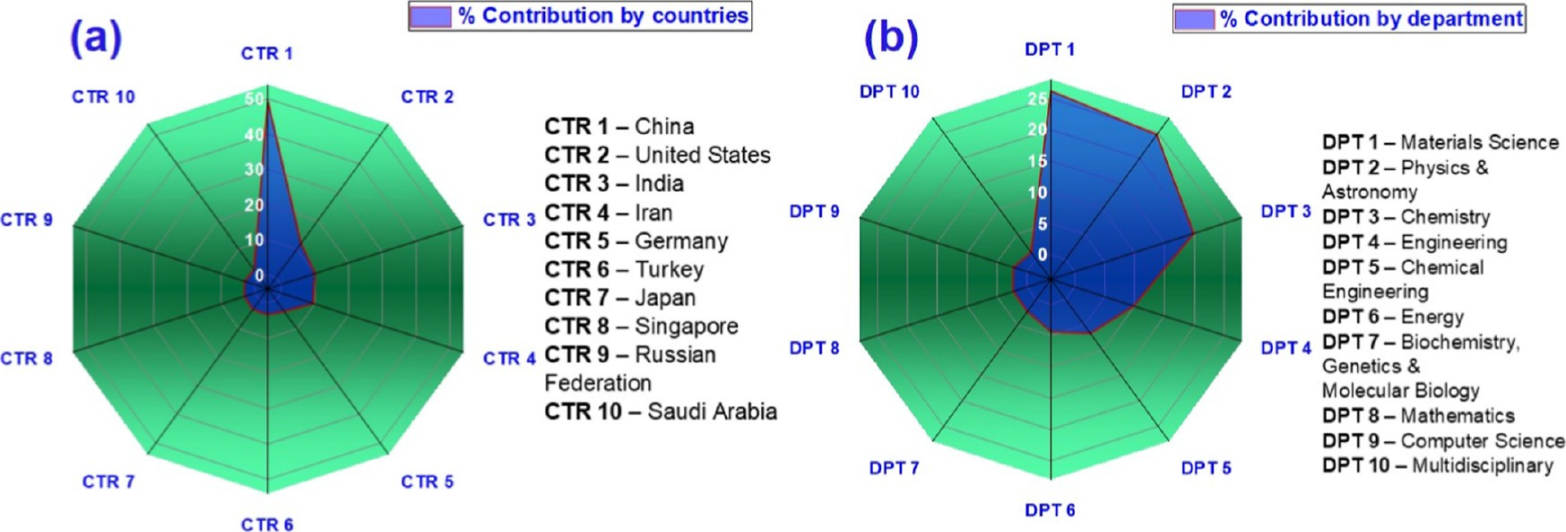
Global knowledge radar: (a) map of contributions by pioneering nations (CTR) and (b) portrait of the global scientific ecosystem by departments (DPT) from the Scopus database.

### 3.3 Hotspots and Impact Metrics

#### 3.3.1 Early Theory

Table 1 shows fundamental publications on boron between 1987 and 2000, which formed the basis for the later development of borophene. The most cited work is that of Strout et al. (2000, 229 citations, J. Phys. Chem. A)^24^, on the structure and stability of B_12_N_12_, seminal for the theoretical study of boron nanostructures. Next comes Michel et al. (1987, 83 citations, Appl. Phys. Lett.),^207^ who dealt with ion implantation and diffusion of boron in semiconductors, highlighting its technological relevance. The studies of Boustani et al. (2000, 58 citations;[Bibr ref18] 1998, 53 citations^19^) introduced theoretical predictions of boron quasicrystals and nanotubes, guiding the experimental search for new allotropic forms. Other important contributions include Salm et al. (1997, 52 citations)^20^ on boron-doped polysilicon, Collart et al. (1998, 42 citations)^21^ on low-energy ion implantation, and Takeuchi et al. (1997, 37 citations)^22^ on shallow junction formation, all essential for microelectronics. First-principles investigation of B_2_–B_14_ clusters with a comparative analysis between quasi-planar/cage structures and strong multicenter bonding—essential for subsequent study on boron nanosheets, -tubes, etc.^23^ Together, these works reveal the dual strand of research during the period: applied, focused on electronics, and fundamental, exploring nanostructures. Of particular note is Strout^24^ which marked the transition from the study of bulk boron materials to their nanoscopic forms, a prelude to borophene.

**Table 1:** Key Publications on Boron between 1987 and 2000

document title (1987–2000)	authors	source	year	citations
systematic ab initio investigation of bare boron clusters: determination of the geometry and electronic structures of Bn (*n* = 2–14)	Boustani, I	Physical Review B	1997	636
new quasi-planar surfaces of bare boron	Boustani, I	Surface Science	1997	296
structure and stability of boron nitrides: isomers of B12N12	Strout, D.L.	Journal of Physical Chemistry A	2000	229
implantation damage and the anomalous transient diffusion of ion-implanted boron	Michel, A.E., Rausch, W., Ronsheim, P.A.	Applied Physics Letters	1987	83
boron quasicrystals and boron nanotubes: ab initio study of various B96 isomers	Boustani, I., Quandt, A., Rubio, A.	Journal of Solid State Chemistry	2000	58
boron in ab initio calculations	Boustani, I., Quandt, A.	Computational Materials Science	1998	53
diffusion and electrical properties of boron- and arsenic-doped poly-Si and poly-Ge_*x*_Si_1–*x*_ (*x* ∼ 0.3) as a gate material for Sub-0.25 μm complementary metal oxide Semiconductor applications	Salm, C., Van Veen, D.T., Gravesteijn, D.J., Holleman, J., Woerlee, P.H.	Journal of the Electrochemical Society	1997	52
characterization of low-energy (100 eV to 10 keV)boron ion implantation	Collart, E.J.H., Weemers, K., Gravesteijn, D.J., Van Berkum, J.G.M.	Journal of Vacuum Science and Technology B Microelectronics and Nanometer Structures	1998	42
shallow junction formation by polyatomic cluster ion implantation	Takeuchi, D., Shimada, N., Matsuo, J., Yamada, I.	Nuclear Instruments and Methods in Physics Research Section B Beam Interactions with Materials and Atoms	1997	37

#### 3.3.2 Cluster Chemistry

Table 2 lists fundamental publications on boron between 2001 and 2014, a decisive period for the emergence of borophene. The most cited work is that of Tang and Ismail-Beigi (2007, 858 citations, Physical Review Letters), which demonstrated the stability of planar boron sheets and the competition between two- and three-center bonds, establishing the theoretical foundation of the field. Subsequently, Piazza et al. (2014, 727 citations, Nature Communications)^155^ identified the B_36_ cluster as a stable planar unit, providing decisive experimental evidence for the viability of two-dimensional boron sheets. Li et al. (2014, 333 citations, JACS)^31^ introduced the B_35_ cluster with hexagonal vacancies, anticipating the true structure of borophene. Other studies expanded the research: Liu et al. (2013, 232 citations, Scientific Reports)^25^ investigated growth on metal substrates; Zhao et al.^xx^ explored boron as a dopant in graphene; and theoretical work by Özdoğan et al. (2010, 170 citations),^26^ Er et al. (2009, 151 citations),^27^ and Wu et al. (2011, 146 citations)^28^ predicted structural properties and applications such as hydrogen storage. Thus, these studies show the transition of borophene from theoretical speculation to experimental reality.

**Table 2:** Key Publications on Boron between 2001 and 2014

document title	authors	source	year	citations
novel precursors for boron nanotubes: the competition of two-center and three-center bonding in boron sheets	Tang, H., Ismail-Beigi, S.	Physical Review Letters	2007	858
planar hexagonal B 36 as a potential basis for extended single-atom layer boron sheets	Piazza, Z.A., Hu, H.-S., Li, W.-L., Wang, L.-S.	Nature Communications	2014	727
the B35 cluster with a double-hexagonal vacancy: a new and more flexible structural motif for borophene	Li, W.-L., Chen, Q., Tian, W.-J., Wang, L.-S.	Journal of the American Chemical Society	2014	333
from boron cluster to two-dimensional boron sheet on Cu(111) surface: growth mechanism and hole formation	Liu, H., Gao, J., Zhao, J.	Scientific Reports, 3, 3238	2013	232
local atomic and electronic structure of boron chemical doping in monolayer graphene	Zhao, L., Levendorf, M., Goncher, S., Pasupathy, A.N.	Nano Letters, 13(10), pp. 4659–4665	2013	194
the unusually stable B100 fullerene, structural transitions in boron nanostructures, and a comparative study of α- and γ-boron and sheets	Zdógan, C.O., Mukhopadhyay, S., Hayami, W., Boustani, S.	Journal of Physical Chemistry C	2010	170
DFT study of planar boron sheets: a new template for hydrogen storage	Süleyman, Er., De Wijs, G.A., Brocks, G.	Journal of Physical Chemistry C	2009	151
DFT study of hydrogen storage by spillover on graphene with boron substitution	Wu, H.-Y., Fan, X., Kuo, J.-L., Deng, W.-Q.	Journal of Physical Chemistry C	2011	146

#### 3.3.3 Transition to 2D Boron

Figure 4 shows the technological evolution of research on borophene (“boron sheet” and “boron cluster”) based on the number of articles published over three distinct periods: 1987–2000, 2001–2014, and 2015–2024. The vertical axis represents the number of scientific articles, while the horizontal axis divides this trajectory into three stages of technological maturity: birth, growth, and commercial adoption. In the first stage, between 1987 and 2000, the graph identifies the “birth” phase, characterized by a very small number of publications and the emergence of the first theoretical predictions about the possible structures and properties of boron-based materials. This phase was marked by conceptual studies and great expectations regarding the potential of these materials.^24,29^ The second stage, between 2001 and 2014, represents the “growth” period, when there was a significant increase in the number of published articles. This growth is associated with experimental advances that validate the initial theoretical predictions. It was a key moment in understanding the properties and scientific recognition of the material as a potential candidate for innovative technological applications.^30–32^ Finally, the period from 2015 to 2024 is described as the “commercial adoption” phase, reflecting the maturation of research and the emergence of real-world applications, albeit at an early stage. The consolidation of studies in this phase indicates that borophene is approaching a transition point between laboratory and industrial use, driven by advances in synthesis, characterization, and technological application techniques.^33–35^

**Figure 4:**
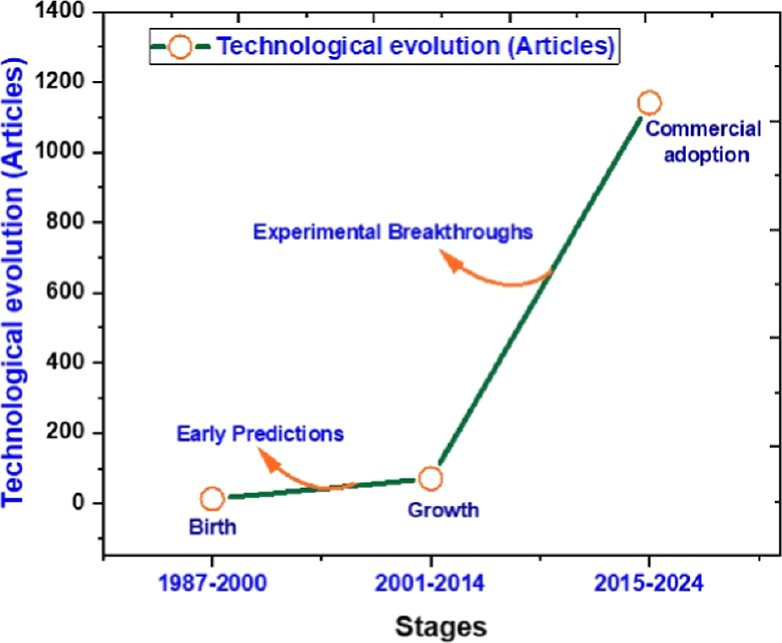
Technological revolution timeline 1987–2024 obtained from the Scopus database.

#### 3.3.4 Precursor Discoveries

The keyword distribution shows that the borophene research field is strongly concentrated in a few core topics, with expansion into complementary areas, as shown in Figure 5. KWD 1, with 17.98%, clearly represents the core of the field, the material itself (“borophene” or “boron sheets”). Next, KWD 2 (17.55%) and KWD 3 (12.97%) reflect theoretical and methodological pillars, such as “density functional theory (DFT)”, “first-principles calculation”, “electronic structure”, or the insertion within “2D materials”. In the intermediate range, KWD 4 (9.59%), KWD 5 (8.61%), and KWD 6 (7.9%) cover specific properties such as “anisotropy” and “superconductivity”, potential applications such as “hydrogen storage” and “sensors”, as well as synthesis methods such as “chemical vapor deposition (CVD).” The lowest-contributing topics, from KWD 7 (7.79%) to KWD 10 (5.72%), correspond to more specialized niches, including “heterostructures,” “doping,” “defects,” and “phase transitions.” Overall, the distribution confirms a pattern typical of emerging fields: a dominant focus on the material, supported by theoretical foundations, followed by properties, synthesis, applications, and a long tail of expanding niche topics.

**Figure 5:**
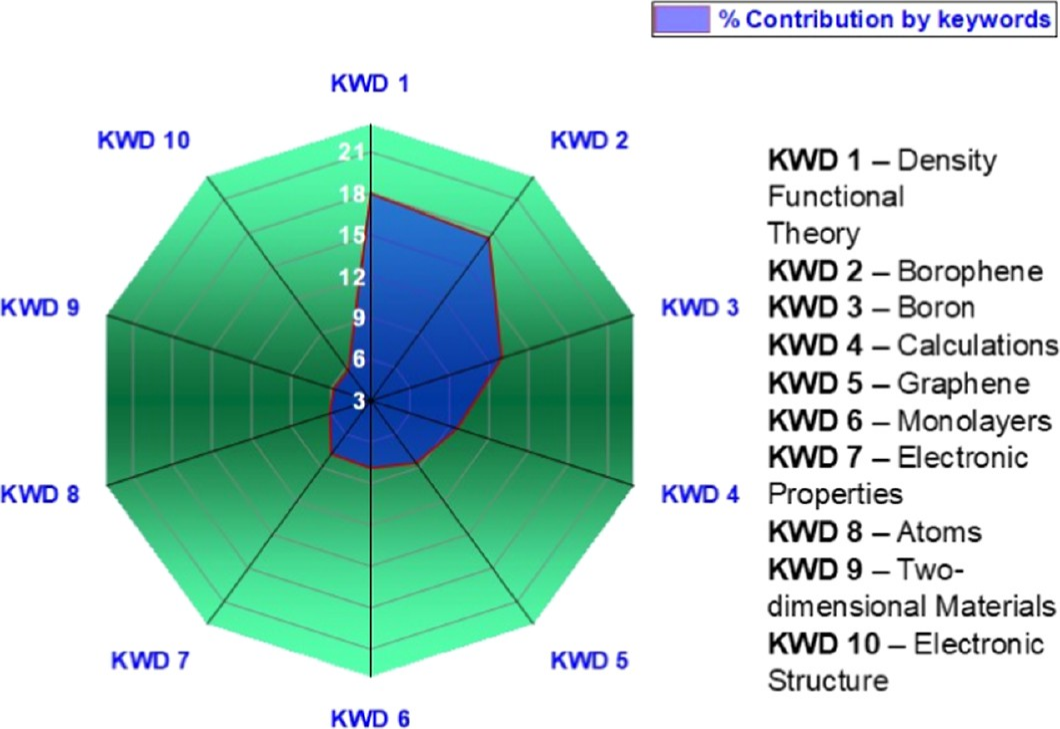
Main keywords distribution in the borophene research field obtained from the Scopus database.

## 4 From Prediction to Experimental Research

### 4.1 Theoretical Foundations

#### 4.1.1 Foundations of Boron Clusters (1987–1999)

Hanley and Anderson in 1988 ^36^ studied the mass spectra of cationic clusters and performed collision-induced dissociation, demonstrating stability at certain sizes, especially B^13+^, and identifying that B^9+^ has a more open structure, explaining its greater reactivity. The geometry of clusters B^1–13+^ was not discussed, but it would be interesting to illustrate the dissociation channels of the larger clusters (Figure 6a). Kawai and Weare in 1991 ^37^ studied the structure and stability of the icosahedral B_12_ cluster using the local density approximation (LDA)-based density functional theory (DFT) to determine the electronic structures. The DFT was developed in the 1960s, which saw in the 1980s strong opposition from the users of traditional ab initio quantum chemical methods. However, widespread application of DFT to clusters began first in the 1990s. They used the Car–Parrinello ab initio molecular dynamics simulation to optimize the geometry, using a simulated annealing approach to find the ground-state configuration of electrons and atoms by heating the system above melting and cooling it slowly or freezing the system into the lowest energy structure. Kato and Tanaka^38^ observed that stable B_*n* (*n* = 4–8) clusters exhibit cyclic and planar character. Kawai and Weare in 1992 ^39^ revealed that B_2_–B_8_^+^, except the dimer, form “magic clusters” with strong multicenter bonds, while B_12_ is metastable and rearranges into more stable open forms, and B_13_ evolves from an icosahedral structure to capped hexagons with high symmetry (*C*_3_ *v*). Kato and Yamashita^40^ confirmed the predominance of cyclic geometries with a central atom in neutral and cationic B_*n* (*n* = 2–12) clusters, as shown in (Figure 6b). The electronic wave functions are efficiently updated as soon as the cores of boron atoms in B_12_ move, and the corresponding forces are calculated. The total energy is calculated using the local density approximation. Their calculation found a new B_12_ structure with a 0.62 eV lower energy than the relaxed icosahedron (Figure 6c–g). Unlike the icosahedron, this new structure is open with *C*_*s*_ symmetry. This open structure possesses a hexagonal pyramid sharing atoms with pentagonal pyramids. The coordination number of the atom varies from 3 to 6, and the number of bonds (Nb = 27) is less than that of the icosahedron (Nb = 30). Nevertheless, because it has fewer nonbonding orbitals, this structure is more stable than the icosahedron. This open structure is confirmed two years later by Boustani,^41^ as shown in Figure 7a–f^42^ and 8a–f.

**Figure 6:**
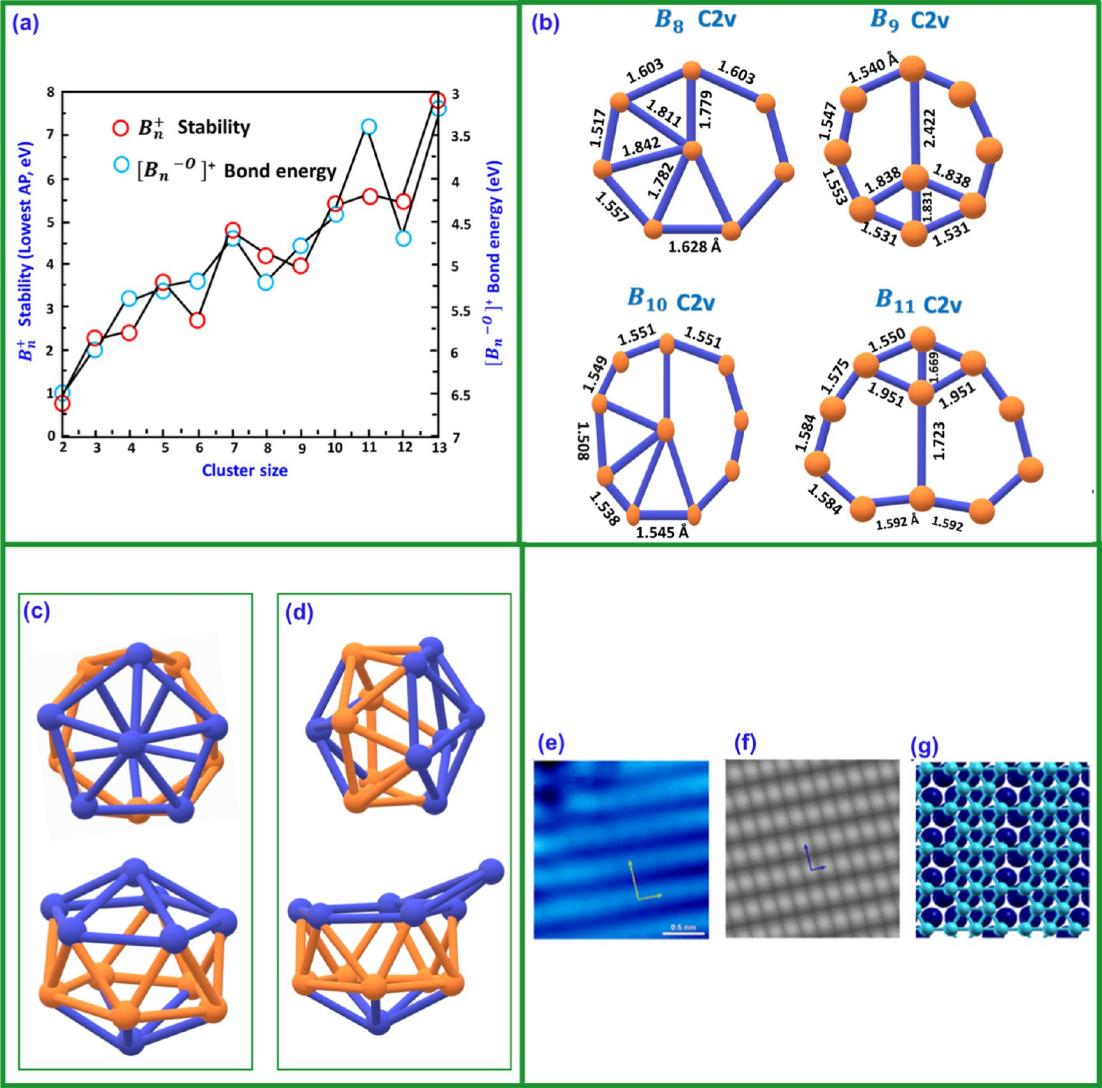
(a) Stabilities compared with estimated [Bn-O] + bond energies. Reproduced with permission from ref 36 Copyright (1988), AIP Publishing. (b) Geometries (in Å) of the most stable B_8_–B_11_ clusters optimized at the HF/3-21G level. Upon ionization, the structures exhibit minimal changes, maintaining primarily planar and cyclic configurations. Reproduced with permission from ref 40 Copyright (1992), Elsevier. (c) Optimized icosahedral B_12_ and (d) open structure found by the simulated annealing method. Reproduced with permission from ref 37 Copyright (1991), AIP Publishing. (e) STM image of the β_12_ phase taken at *V* = −0.7 V, *I* = 1 nA. (f) DFT-simulated image of β_12_ borophene. (g) Top view of the relaxed structure of the β_12_ phase on Au(111). In the background, gold atoms are depicted in a dark blue color, while light blue represents boron atoms. Reproduced with permission from ref 43 Copyright (2024), American Chemical Society.

**Figure 7:**
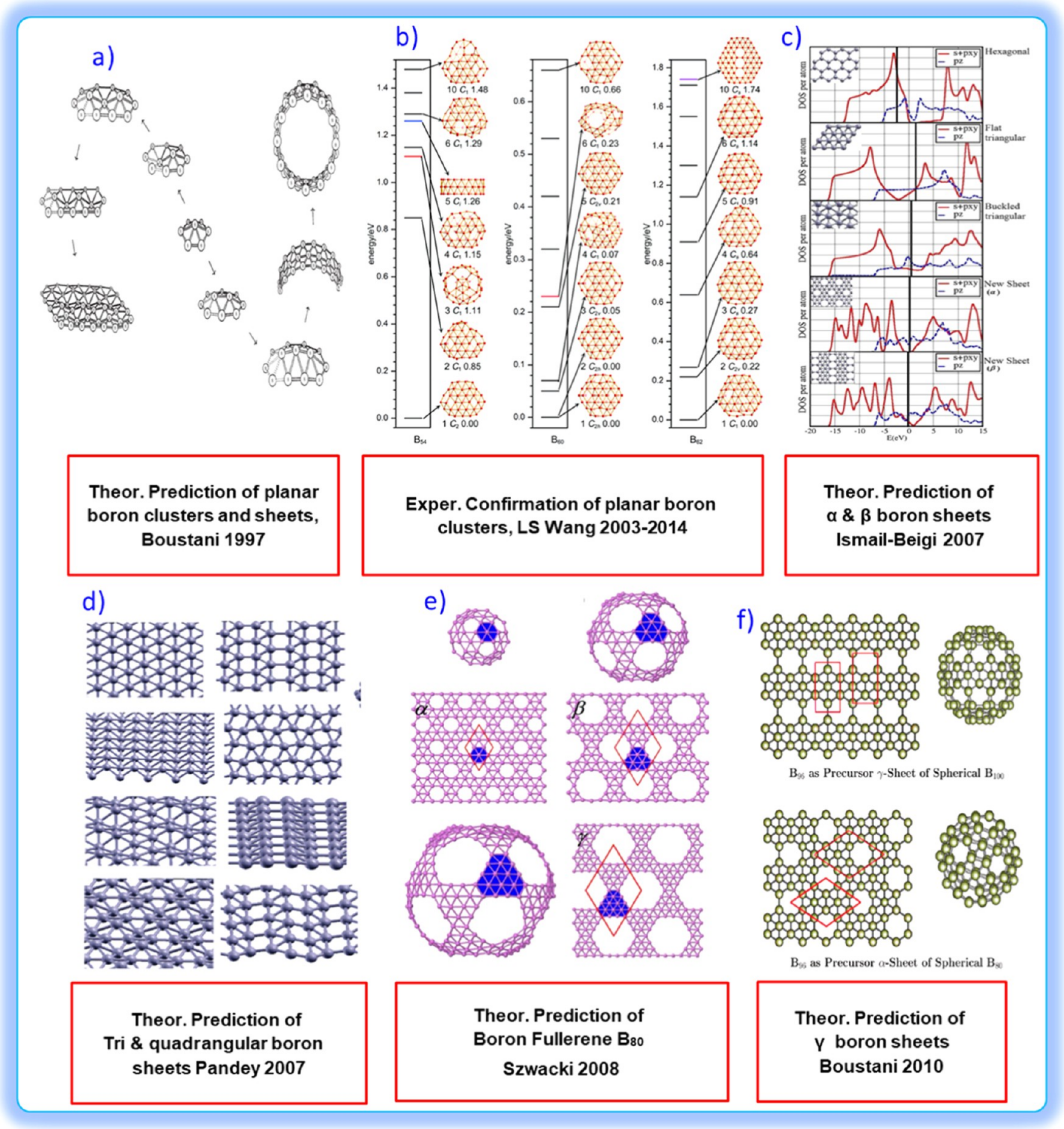
(a) Theoretical and experimental development of BPh. Schematic diagram of the boron cluster growth according to the “Aufbau principle”. Reproduced with permission from ref 55 Copyright (1997), American Physical Society. (b) Configurational energy spectra at the PBE0/6-311+G* level B_54_; B_60_ and B_62_ at PBE0/6-311+G. Reproduced with permission from ref 56 Copyright (2020), Wiley. (c) Projections onto in-plane (s, p_*x*_, p_*y*_; solid red) and out-of-plane (p_*z*_; dashed blue) orbitals. Thick vertical lines indicate the Fermi level EF (Gaussian broadening: 0.3 eV). Reproduced with permission from ref 57 Copyright (2013), American Physical Society. (d) Boron sheets: hexagonal, graphene-like, idealized, and buckled. Reproduced with permission from ref 58 Copyright (2007), American Chemical Society. (e) Three members of the fullerene family are shown: B_80_, B_180_, and B_300_. Reproduced with permission from ref 59 Copyright (2008-License: CC BY 2.0), Springer Nature. (f) B_96_ clusters as precursors for the γ-sheet of the B_100_ sphere and for the R-sheet of the B_80_ sphere. Reproduced with permission from ref 26 Copyright (2010), American Chemical Society.

**Figure 8:**
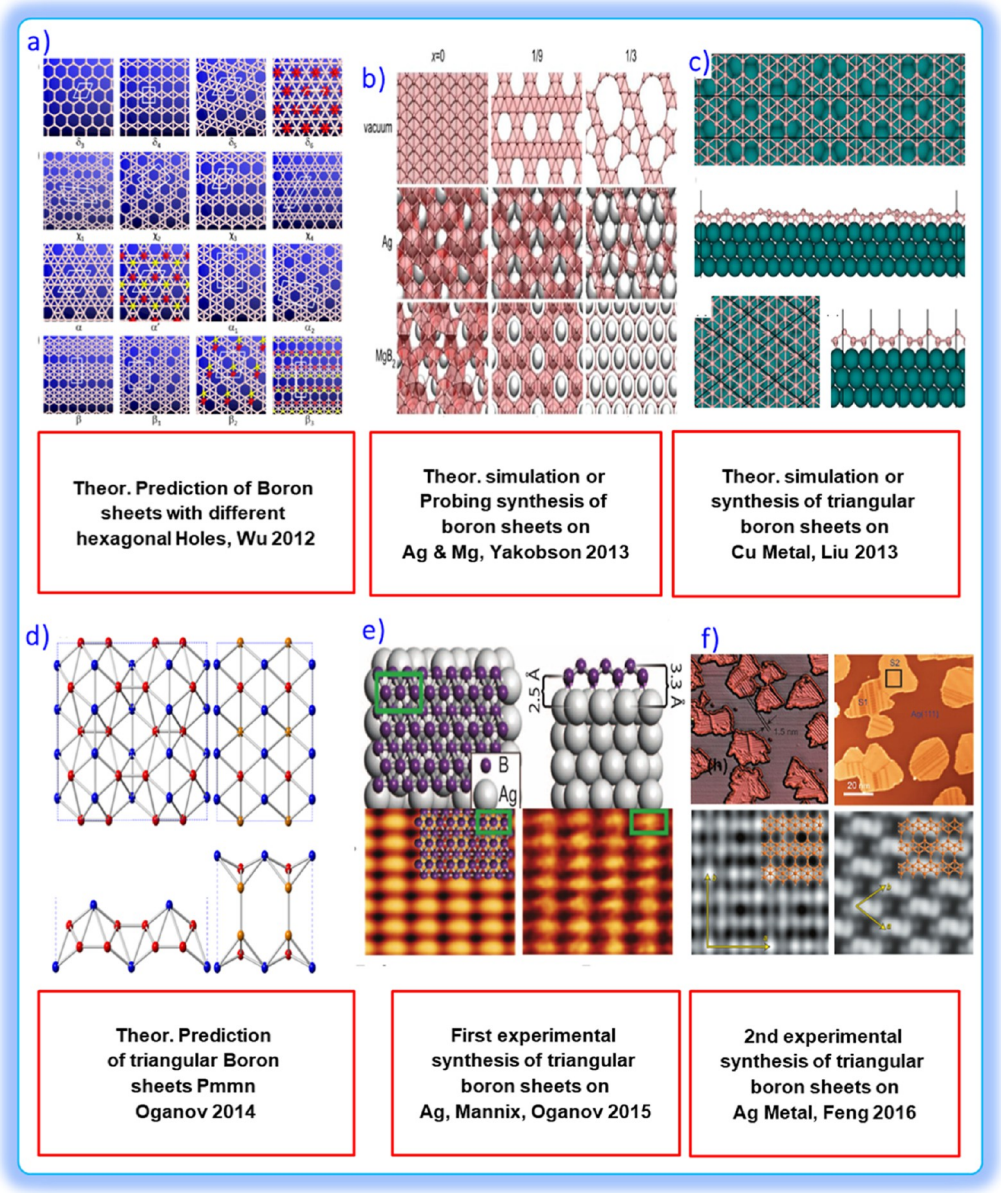
(a) Low-energy structures of (a) δ-, (b) χ-, (c) R-, and (d) β-type boron monolayers. Red and yellow spheres indicate boron atoms displaced outward or inward, forming buckled sheets. Reproduced with permission from ref 62 Copyright (2012), American Chemical Society. (b) Atomic structure of BPh (*x* = 0, 1/9, and 1/3) in vacuum and on substrates: Ag(111)- and Mg-terminated MgB_2_(0001) surfaces. Reproduced with permission from ref 60 Copyright (2013), Wiley. (c) Atomic structures of two representative boron monolayers on the Cu(111) surface. Reproduced with permission from ref 25 Copyright (2013-License: CC BY-NC-ND 3.0), Nature. (d) Projections of the 2 × 2 × 1 supercell of the *Pmmn* and *Pmmm* structures of boron along the [001] and [100] directions, with the nonequivalent atomic positions highlighted in different colors. Reproduced with permission from ref 61 Copyright (2014), American Physical Society. (e) Computational prediction of the BPh structure and electronic properties. Reproduced with permission from ref 63 Copyright (2015), Science. (f) Structure models of S1 and S2 phases of BPh based on DFT calculations. Reproduced with permission from ref 64 Copyright (2023), Elsevier.

Tang and Liu in 1993 ^44^ introduced conjugated carbon–boron polyhedra, allowing the prediction of the geometry, stability, and structural properties of borophene. In 1994, Boustani^41^ demonstrated that quasi-planar B_*n* ^+^ (*n* = 2–14) clusters are more stable than 3D structures, challenging the icosahedral dogma. In 1995, Boustani^45,46^ used ab initio and DFT methods to show that neutral clusters exhibit convex or quasi-planar structures, also predicting elementary clusters with ∼90 atoms. Quasi-planarity was confirmed by Ricca and Bauschlicher^47^ in B_*n* ^+^ (*n* ≤ 14) clusters. Kramer and Boustani in 1996 ^48^ studied the α-rhombic unit of boron and derived clusters, simulating the interaction of two icosahedral clusters, resulting in tubular B24 and in models based on B21, elucidating structural patterns for icosahedral-derived borophene. In 1997, Boustani et al.^23,49–51^ identified pentagonal (B6) and hexagonal (B7) pyramids as fundamental building blocks, suggesting formation mechanisms for borophene or 2D boron nanotubes. Slanina and Lee^52^ studied clusters B_2_–B_8_^+^, B_12_, B_13_ and B_32_, evidencing multicentric bonds, geometric flexibility, and stability in open or capped hexagonal forms. Boustani and Alonso^53^ analyzed B_32_, highlighting the stability resulting from the balance between curvature and elimination of dangling bonds. Park and Cho^54^ investigated electronic and structural properties of B_2_–B_8_ and B_12_, providing a reliable basis for modeling and predicting borophene properties.

Liu et al.^60^ reveal through first-principles calculations the energy pathways and fundamental conditions that govern the synthesis of two-dimensional boron, elucidating the atomic mechanisms that enable the stable formation of 2D sheets (Figure 8a,b). Liu et al.^25^ elucidated the growth mechanism that transforms boron clusters into a two-dimensional sheet on the Cu(111) surface, revealing how atomic diffusion and coalescence lead to the formation of characteristic holes in the boron-containing lattice (Figure 8c). Zhou et al.^61^ presented the discovery of a two-dimensional boron allotrope with semimetallic behavior that harbors massless Dirac Fermions, revealing a promising 2D material for advanced electronic applications (Figure 8d–f).

#### 4.1.2 Main Studies on Boron Clusters (2000–2010)

Between 2000 and 2010, several studies established the theoretical foundations of borophene, as shown in Figure 9. In 2000, Fowler and Ugalde^65^ examined B_13_ clusters in different charge states via DFT, showing that planar or quasi-planar structures are more stable than three-dimensional ones, with π delocalization conferring aromaticity, especially in B_13_^+^. Zhu and Henley^66^ constructed coordination-tuned classical potentials, connecting geometry and atomic stability, as shown in Figure 9a–c. In 2001, Aihara^67^ confirmed the high aromaticity of the B_13_^+^ cluster, reinforcing the relationship between planar stability and electronic resonance. Cao and Zhou^23^ identified multiple stable structures of B_7_, B_10_, and B_13_, highlighting the preference for planar geometries. Peeters and Doren^68^ studied B_12_ clusters in graphite, showing how boron defects interact with extended lattices. Between 2002 and 2004, Zhai and Wang^69^ investigated B_5_^–^ and B_5_, evidencing planar *C*_2_ *v* structures with fully delocalized π bonding. Li and Jin^70^ confirmed the stability of B_5_ clusters at different charges, highlighting multicentric bonds and σ and π aromaticity. Jin and Li,^71^ Zhai and Wang,^72–74^ and Chacko et al.^75^ showed that extreme planarity, sp^2^ hybridization, and double aromaticity are central principles in the stability of boron clusters. Studies by Alexandrova et al.^76,77^ and Lau et al.^78^ showed transitions from small clusters to tubular structures, establishing building blocks for borophene and boron nanotubes. From 2005 to 2006, Gillery et al.,^79^ Linguerri et al.,^80^ Marques and Botti,^81^ Kiran and Wang,^82^ Molina et al.,^83^ Aihara and Ishida,^84^ and Alexandrova et al.^85^ reinforced the importance of aromaticity, electronic delocalization, and modularity for structural stability and electronic properties. Studies by An et al.,^86^ Lau and Pineda,^87^ Cabria and Alonso,^88^ and Kunstmann^89^ investigated sheets and nanotubes, revealing mixed metallic/covalent bonding, corrugated surfaces, and planar-to-tubular transitions. Between 2007 and 2009, Oger et al.,^90^ Satpati and Sebastian,^91^ Zubarev and Boldyrev,^92^ Pan et al.,^93^ Zubarev and Boldyrev,^94^ Sergeeva et al.,^95^ Zhao et al.,^96^ Kiran et al.,^97^ Atiş et al.,^98^ Johansson,^99^ and Bean and Fowler^100^ have detailed B_12_–B_24_ cluster evolution, multiple aromaticity, ring currents, and 2D → 3D transition. Szwacki et al.^101^ introduced borozene, an aromatic analogue of benzene. Ohishi et al.^102^ demonstrated structural control of B_12_H_*n*_^+^ clusters via the number of hydrogens, as shown in Figure 9d. In 2010, Tai et al.,^103^ Slough et al.,^104^ Huang et al.,^105^ Jiménez-Halla et al.,^106^ Forte et al.,^107^ and Ohishi et al.^108^ demonstrated multiple aromaticity, double concentric π-aromaticity, unique rotational mechanisms (“Wankel engine”), and semiconductor properties, consolidating the fundamental structural and electronic principles for the formation of two-dimensional boron sheets (borophene) and their applications in nanotechnology, as shown in Figure 9e–k.

**Figure 9:**
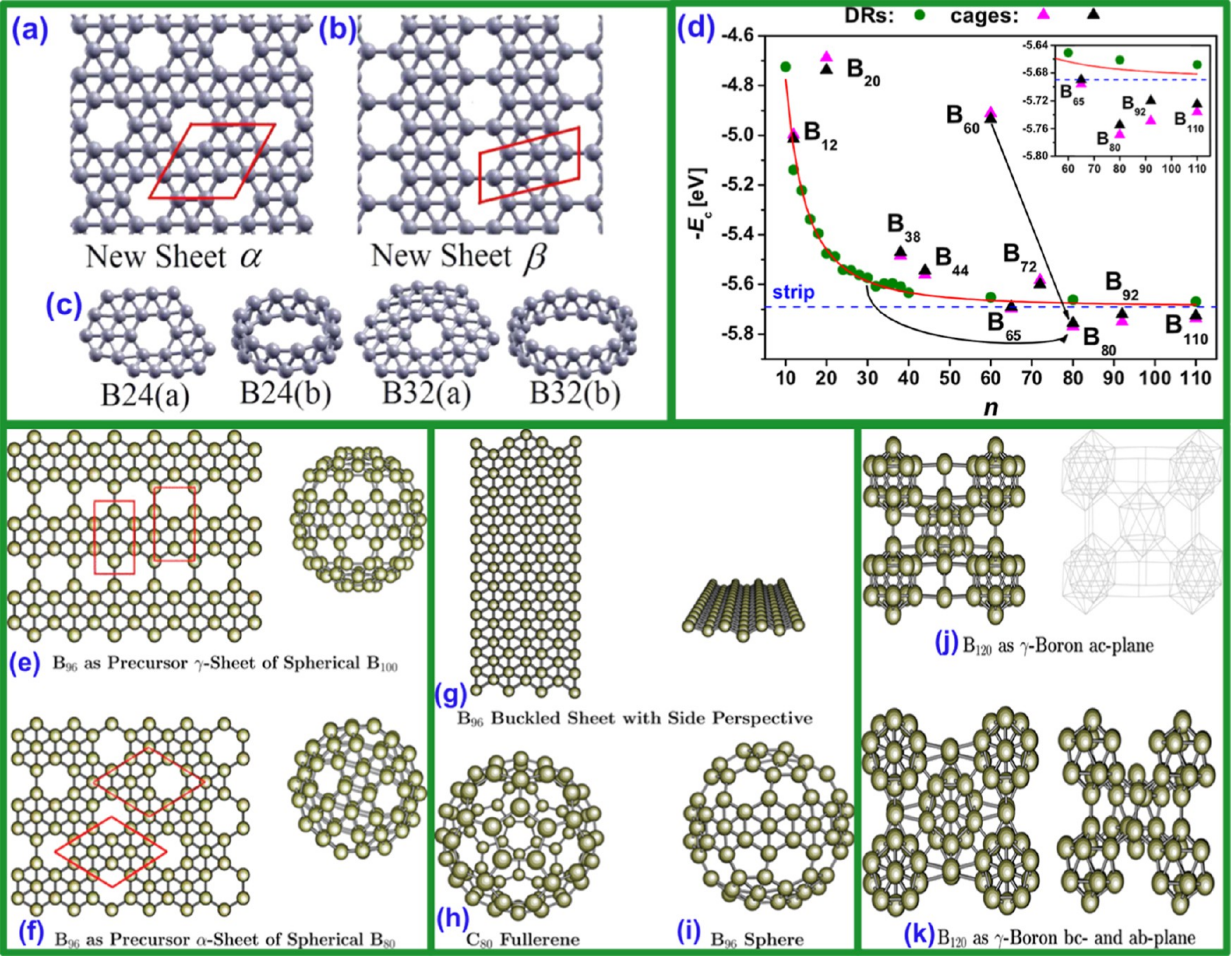
(a,b) Two examples of our BS (top view). Red solid lines show the unit cells. (c) Four boron clusters: B_24_(a) and B_32_(a) are clusters with hexagonal holes; B_24_(b) and B_32_(b) are the double-ring clusters. Gray balls are boron atoms, and gray “bonds” are drawn between nearest neighbors. Reproduced with permission from ref 57 Copyright (1991), AIP Publishing. (d) Cohesive energy per atom in B_*n*_ clusters: circles (double rings), triangles (cages, QE black/GAUSSIAN03 magenta). Blue line: infinite strip. Arrows: energy gain via hexagon reinforcement (B_60_ → B_80_) and crossed rings (B_30_ DR → B_80_). Inset: relative *E*_(c)_ for B_65_, B_80_, B_92_, B_110_. Reproduced with permission from ref 109 Copyright (2007), AIP Publishing. (e) B_96_ clusters as precursors for the γ-sheet of the B_100_ sphere and for the (f) α-sheet of the B_80_ sphere. (g) B_96_ cluster of the buckled sheet with a side perspective. (h) Carbon C80 fullerene as a scaffold for generating the (i) boron B_96_ sphere. (j) B120 (9 × B_12_ + 6 × B_2_) cluster, as a cut of the γ-B_28_ orthorhombic solid boron, is composed of nine boron icosahedra and six boron pairs, (k) viewing the perspective of the planes *ac*, *bc*, and *ab*. Reproduced with permission from ref 26 Copyright (2010), American Chemical Society.

#### 4.1.3 Progress in Borophene Development (2011–2024)

In 2011, several studies expanded our understanding of the structure and properties of boron clusters, focusing on borophene, a two-dimensional boron material, as shown in Figure 10a,b. Martínez-Guajardo et al.^110^ described the fluxionality of the B13+ cluster, showing that the inner triangle is connected to the peripheral ring by delocalized bonds, allowing nearly free rotation of the inner core. Bean et al.^111^ investigated magnetically induced ring currents in the boron buckyball B80, observing similarity to carbon buckyballs, but with a higher current density. Boustani, Zhu, and Tománek^112^ studied the structural stability and vibrational spectra of small boron clusters, observing that the most stable isomer of B19 is two-dimensional as shown Figure 10b. Romanescu et al.^113^ investigated aluminum-doped clusters, showing how doping can modulate the planarity and stability of boron clusters. Recent studies have explored the structure, stability, and electronic properties of 2D boron sheets. March and Rubio^114^ analyzed buckled benzene and graphene analogues, highlighting π-delocalization and potential applications in catalysis, lubrication, and electronic/photonic devices. Sergeeva et al.^115^ and Li et al.^116^ investigated anionic and Al-doped clusters, showing planarity/quasi-planarity and dual aromaticity (σ and π), fundamental for the stability and design of borophenes. Galeev et al.^117^ demonstrated that Al doping generates unique structures supported by ionic interactions and aromaticity. Shin et al.^118^ proposed a diatomic model to predict structural patterns and binding energies in boron clusters, consolidating theoretical foundations for 2D boron materials.

**Figure 10:**
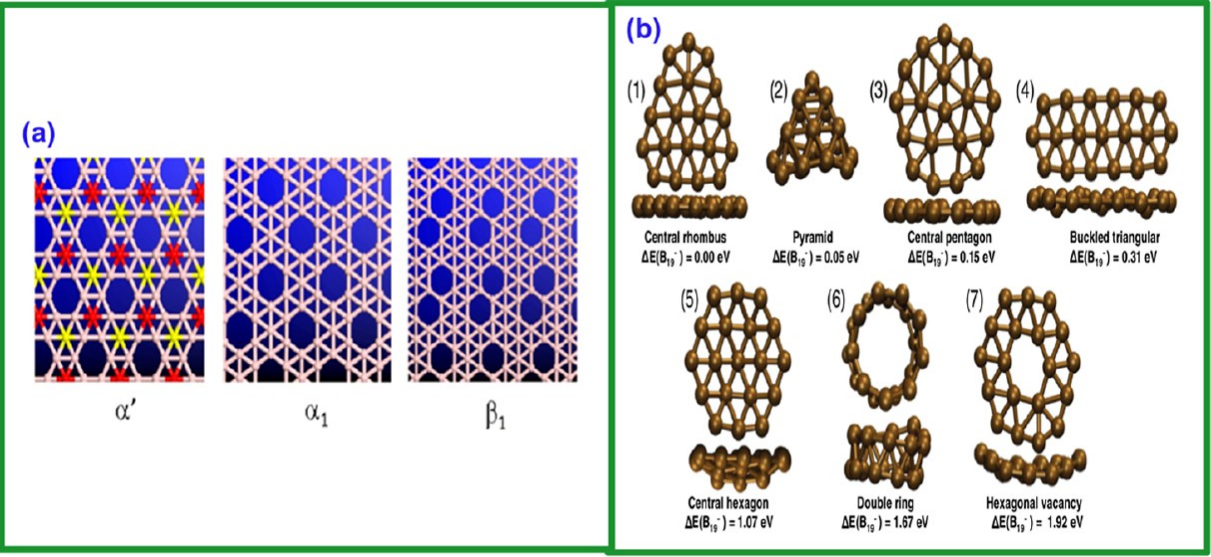
(a) 2D boron monolayer sheets. Reproduced with permission from ref 62 Copyright (2012), American Chemical Society. (b) Equilibrium geometries and zero-point-corrected total energy differences of B^–19^ anion isomers. Selected structures show varying planarity in top and side views. Reproduced with permission from ref 137 Copyright (2011), American Physical Society.

In 2012, Zhang, Zhang et al.^119^ explored how electromagnetic radiation can induce rotation of molecular structures such as the B_13+_ double ring, as shown in Figure 10b. Gonzalez Szwacki and Tymczak^120^ demonstrated that the quasi-planar structures of B_12_H_*n*_ and B_12_F_*n*_ are energetically more favorable. Tai et al.^121^ analyzed the stability of cationic boron clusters B_*n*+_ (*n* = 2–20), finding stability in both 2D and 3D forms. Li et al.^122^ investigated boron rings doped with transition metals, such as Rh©B_9–_ and Ir©B_9–_, revealing *D*_9*h*_ symmetry and electronic stability. Li et al.^123^ described H_2_B_*n*–_ clusters (*n* = 7–12) with delocalized σ and π bonds, analogous to polyenes, suggesting π-conjugated molecular wires. Li et al.^124^ showed that the aromatic molecular wheel *D*_10*h*_-V©B_10–_ is unstable; the most stable form of VB_10–_ is a singlet (C2) “boat” with V coordinated to the quasi-planar B_10_. Galeev et al.^125^ investigated metal-doped boron clusters such as NbB_10–_ and TaB_10–_, showing stability in wheel-like structures. Romanescu et al.^126^ observed iron-doped boron clusters (*C*_8*v*_-Fe©B_8–_ and *D*_9*h*_-Fe©B_9–_), both doubly aromatic (σ + π) with delocalized interactions between Fe and the boron ring. Romanescu et al.^127^ showed that the neutral clusters B_11_, B_16_, and B_17_ are planar or quasi-planar, similar to the corresponding anionic ones. Galeev et al.^128^ studied carbon-doped clusters (CB_9–_ and C_2_B_8–_), finding distorted wheel-like structures with π aromaticity and σ antiaromaticity. Yuan and Cheng^129^ reported that B_20_ and B_142+_ are magic number clusters with double rings and double aromaticity, with B_142+_ being more stable than the quasi-planar by 1.2 eV. BC et al.^130^ showed that B_19_–B_24_ clusters oscillate between planar and 3D ring structures depending on the even/odd number of atoms. Sergeeva et al.^131^ analyzed B_22–_ and B_23–_, finding quasi-planar or heart-shaped structures with delocalized π-bonds similar to polycyclic aromatic hydrocarbons. Piazza et al.^132^ revealed that B_21–_ is quasi-planar with delocalized σ- and π-bonds, similar to B_19–_. Tai et al.^133,134^ revisited B_14_–B_20_, showing a 2D → 3D transition in neutral (tubular) B_20_, while B_20–/2–_ anions are planar, doubly cyclic, and fluxional. Cheng^135^ showed that neutral B_14_ is a planar, highly aromatic, and stable capsule, challenging previous models. Li et al.^136^ confirmed that core–shell structures (fullerenes) are favored for B_80_, B_101_, and B_103_, warning about the proper use of density functionals in boron nanomaterials.

In 2013, Pham et al.^138^ explored transitions between 2D and 3D forms of boron clusters, and Bai et al.^139^ investigated B_12_Au- and B_13_O-clusters, showing that they are aromatic compounds with six π electrons. Tai et al.^140^ predicted the planar structure of B_202–_ with a circular circumference, correlating the electronic structure to Bessel functions. Liu, Gao, and Zhao^25^ investigated the stability of boron sheets on the Cu(111) substrate, encouraging the experimental synthesis of borophene. Lu and Li^141^ proposed three-chain boron cages B6*n* + 14 (*n* = 1–12), with D3/C3 symmetry, constructed by fusing boron double half-rings, suggesting bottom-up synthesis routes to boron fullerenes, such as B_80_. Romanescu et al.^142^ investigated MB_9–_ (M = V, Nb, Ta), showing that V fits into B_9_ forming a planar structure (*C*_2*v*_/*D*_9*h*_), while Nb and Ta cause slight distortions due to the Jahn–Teller effect. Popov et al.^143^ analyzed B_24–_, a quasi-2D isomer with a tendency toward 3D pentagonal units; the quasi-planar isomer contributes to the photoelectron spectrum, presenting peripheral σ bonds and internal π bonds (6c–2e). Romanescu et al.^144^ highlighted that borometallic clusters (M©B_*n*_ and M©B_*n*–_) maintain dual aromaticity (σ + π) and high electronic stability, offering potential for large-scale synthesis and new functional nanomaterials.

In 2014, Shinde and Shukla^145^ studied the optical absorption of aluminum clusters, while Zhai et al.^42^ reported the structure of B_40–_, similar to fullerene, as shown in Figure 11a. Cervantes-Navarro et al.^146^ investigated the fluxionality of the B_19–_ cluster, and Piazza et al.^147^ identified isomers of B_25–_. Chen et al.^148^ investigated aromatic boron clusters B_36_, providing molecular models for the formation of borophene. Popov et al.^149^ studied CoB_12–_ and RhB_12–_, which exhibit crescent-shaped structures with quasi-planar B_12_ coordinating the metal, exhibiting multicentric σ and π bonds, with potential catalytic sites, as shown in Figure 11b. Duong et al.^150^ showed that B_27+_ forms a stable tubular aromatic triple hollow cylinder, while anions/dianions favor quasi-planar structures. Wang^151^ revealed that small boron clusters are planar or quasi-planar; B_36_ with a central hexagonal hole serves as a unit for borophene, and neutral B_40_ forms a capsule (“borosphere”). Pham et al.^152^ analyzed B_2*n*_ boron tubes (*n* = 10–14) as stable hollow cylinders with tubular aromaticity (4 N + 2 M rule). Fa et al.^153^ studied MB40 (M = Li, Na, K, Ba, Tl), showing endohedral/exohedral dopant preferences and effects on stability. Tai et al.^154^ showed disk aromaticity in B_30_, similar to B_202–_ and B_19–_. Piazza et al.^155^ confirmed B_36–_ as a quasi-planar cluster with a hexagonal vacancy, the basis for 2D sheets. Sergeeva et al.^156^ showed that planar/quasi-planar clusters with σ and multicenter-2e bonds exhibit fluxionality and potential for borometallics and nanomaterials. Li et al.^157^ identified the first chiral boron [B_30_]-cluster, a quasi-planar cluster with a degenerate hexagonal hole. Tandy et al.^158^ demonstrated that compact B_12_ clusters are stable for *N*_atom_ > 200, while capsules and random structures predominate for 24 < *N*_atom_ < 200. Li et al.^31^ showed that boron lattices with hexagonal vacancies (B_36_, B_35–_) are stable, allowing borophenes with variable hole density and triple π aromaticity. Lv et al.^159^ predicted a highly symmetric, energetically favorable fullerene B38 with high double aromaticity. Moreno et al.^160^ studied B_182–_, a quasi-planar and fluxional dianion with double σ and π aromaticity acting as a “Wankel engine.”

**Figure 11:**
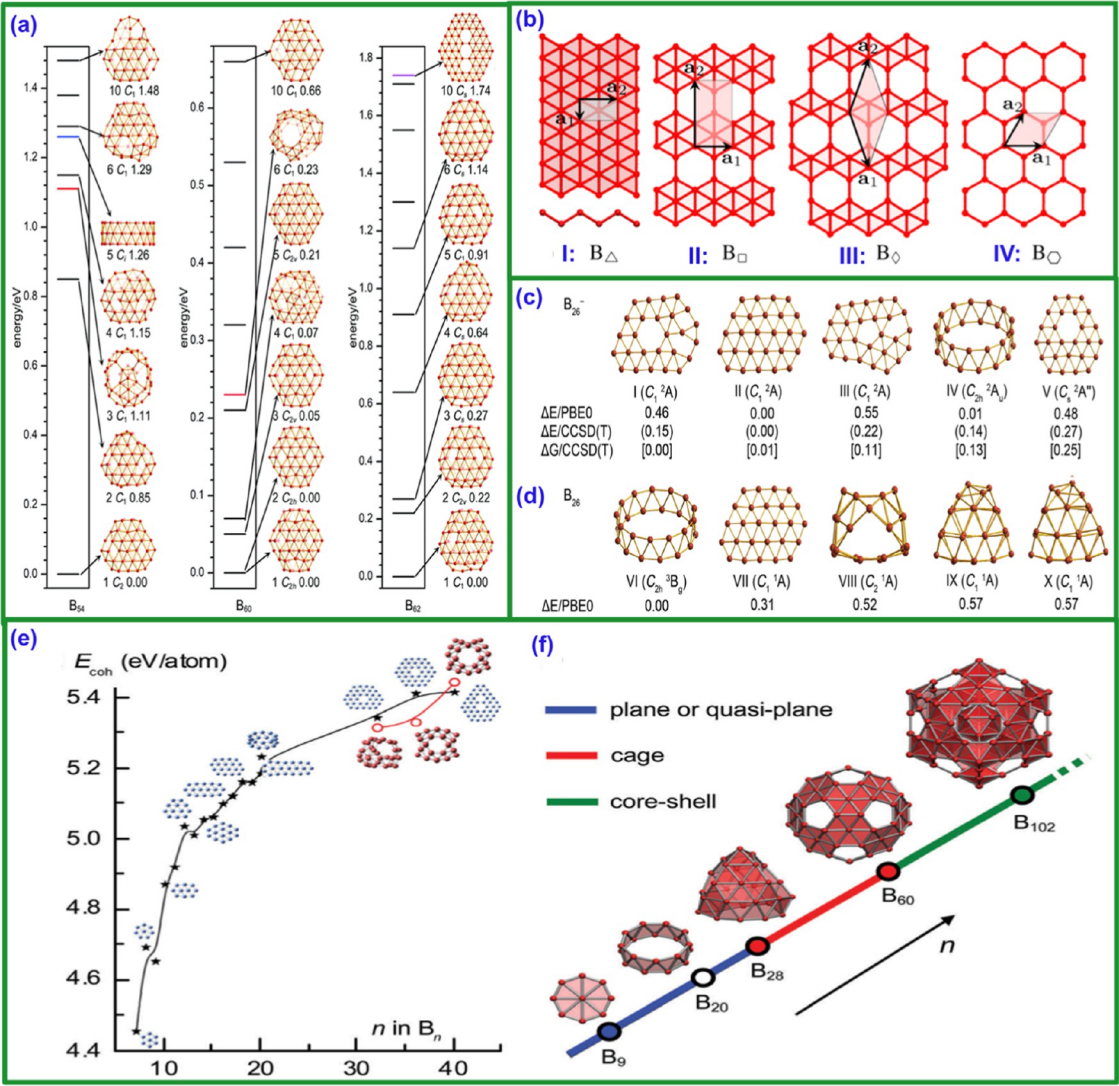
(a) Configurational energy spectra of B_54_, B_60_, and B_62_ at PBE0/6-311+G, relative to the global minimum. Reproduced with permission from ref 56 Copyright (2020), Wiley. (b) Atomic structure model of different borophene phases. (I–IV) Reproduced with permission from ref 184 Copyright (2016), American Chemical Society. Top low-lying B_26_^–^ and (c) neutral isomers with relative energies at PBE0 and (d) CCSD­(T)//PBE0, including ZPE and Gibbs free energy corrections at 460 K. Reproduced with permission from ref 179 Copyright (2017), Elsevier. Boron cluster stability: (e) cohesive energies of neutral B_*n*_ (*n* = 7–40) at PBE0, showing planar/double-ring (*n* = 20) and cage (*n* = 32, 36, 40) structures, with (f) preferred conformations evolving from planar to cage-like and core–shell. Reproduced with permission from ref 185 Copyright (2022), American Chemical Society.

In 2015, Lv et al.^161^ explored the stabilization of borophene structures with transition metals. Martínez-Guajardo et al.^162^ investigated the dynamics of borospherene B_40_, with a cage structure, while Chen et al.^163^ introduced new chiral borospherene species, as shown in Figure 11e,f.^164^ Chen et al.^165^ identified B_39–_ as a 3D chiral borospherene, fluxional above room temperature. Zhao et al.^166^ revealed the B_28_ cage, composed of B_12_ units. Reber and Khanna^167^ analyzed boron clusters with Mg, observing improvements in stability. Xu et al.^168^ identified planar B49 with a double-hexagonal vacancy and proposed a 2c–2e and 3c–2e bond distribution model to explain the stability of planar clusters and boron cages. Rahane and Kumar^169^ reported nearly planar bowl-shaped B_84_ with hexagonal holes and dynamic stability due to multicenter bonds. Tai and Nguyen^170^ showed that neutral B_26_–B_27_ are tubular, while B_28_–B_29_ are nearly planar; anions favor 2D shapes, suggesting a general growth mechanism. Li et al.^171^ investigated B_27–_, identifying a 2D global minimum with a triangular lattice and tetragonal defect, as well as isomers with hexagonal vacancies, a typical feature of medium-sized clusters.

In 2016, Tai, Lee, and Nguyen^172^ identified cages with octagonal holes for B_420/+_, while Tai and Nguyen^173^ revealed the B_44_ structure, with hexagonal and nonagonal holes. Moradi, Vahabi, and Bodaghi^174^ studied NH_3_ adsorption on B_40_, suggesting applications in sensors. Liu and Lukose^175^ reviewed boron clusters, emphasizing their unique structures. Li et al.^176^ showed that B_29–_ is quasi-planar (“stingray”), while neutral B_29_ is borospheric, illustrating the 2D → 3D transition and aromaticity patterns in boron clusters.

In 2017, Sai et al.^177^ showed that large B_*n*_ (*n* = 46–50) exhibit diverse structural motifs, indicating pathways to 2D boron sheets with hexagonal holes. Chen et al.^178^ and Luo et al.^179^ evidenced that hexagonal vacancies and delocalized π-electrons control the stability and evolution of 2D clusters, as shown in Figure 11c,d. Nagare et al.^180^ demonstrated that shape and dimensionality determine optical responses and polarizabilities, which are fundamental for functional borophenes.

In 2020, Shi, Kuang, and Lu^181^ explored lithium-doped boron clusters, revealing highly stable structures. Ghosh and Jana^182^ investigated the B_13+_ cluster as a molecular motor, with peripheral ring rotation.

In 2022 Chkhartishvili^183^ proposed a diatomic model for B_*n*_ (*n* = 1–15), correlating energies and bond lengths with experimental spectra, providing key parameters to understand cohesion, growth, and electronic properties of 2D borophene. These studies continue to solidify the theoretical foundations for the design and understanding of borophenes and also open new possibilities for the manipulation and application of boron-based materials.

### 4.2 Transition from Computational to Experimental Methods

Predicting the different formations of borophene depends heavily on the computational method employed, leading to significant variations in relative stability, hole density, and estimated electronic properties.^186^ Calculations performed with PBE, for example, tend to favor metallic and highly delocalized phases, frequently predicting a wide range of stable sheets due to underestimation of electronic correlation, as observed in pioneering studies of structural modeling of the material.^187^ In contrast, hybrid functionals, such as B3LYP, partially correct this delocalization and can alter the stability order between phases such as β_12_, χ_3_, and δ_6_, also modifying the prediction of bandgaps and bonding motifs, as discussed in refined analyses of the borophene energy landscape.^188,189^ While high-precision electronic correlation correction methods, such as CCSD­(T), are only applicable to reduced-scale models due to their computational cost, they provide the most reliable benchmark for evaluating the robustness of DFT predictions, revealing significant differences in relative energies and typical multicluster binding patterns of boron.^190,191^ These theoretical divergences make experimental validations essential: techniques such as STM and ARPES have demonstrated that certain phases predicted by PBE appear only metastable or substrate-dependent and that kinetic phenomena can prevail over theoretical thermodynamics, as shown in investigations on anisotropy and surface-induced reconstructions.^192^ Theoretical models predicting vacancy density, bonding patterns, and substrate interactions directly influenced experimental synthesis strategies, especially the choice of Ag(111) and Au(111) to stabilize the β_12_ and χ_3_ phases.^193,194^ Nevertheless, discrepancies persist between prediction and observation, as different methods may suggest alternative formations under varying temperatures, deposition rates, or growth conditions, and only controlled synthesis has confirmed the existence of several phases originally predicted only theoretically, culminating in the experimental production reported in fundamental works on borophene synthesis, as shown in Table 3.^63,195^

**Table 3:** Overview of Borophene Synthesis Routes

synthesis method	substrate	phases	sample size	reproducibility	ambient stability	refs
MBE (UHV deposition of B on Ag)	Ag(111)	β_12_, χ_3_, mixed	μm-scale islands	high within same group; moderate reproducibility across groups	rapid oxidation upon exposure; requires encapsulation	^63,195^
MBE on Au(111)	Au(111)	β_12_ variants, buckled phases	μm islands	moderate	similar oxidation issues; substrate charge transfer stabilizes some phases	^196^
CVD (γ-B monolayer)	Cu or Al foil	γ-B (layered γ-B)	larger (continuous films reported)	few groups-needs independent replication	reported improved stability when supported on Cu; passivation needed	^197,198^
LPE/wet chemical	Au(111) or solution	few-layer borophene nanosheets (claims)	small flakes	early stage; reports limited	stability depends on solvent and surfactant; commonly oxygen-sensitive	^199,200^

### 4.3 Experimental Breakthroughs

Figure 12 shows the golden age of experimental borophene research (2015–2024), marked by the actual synthesis of the material and the rapid expansion of studies on its properties and applications, as shown in Figure 12a. The seminal work is that of Mannix et al.^63^, who synthesized anisotropic 2D sheets of boron on silver, consolidating decades of theory. Soon after, Feng et al.^195^ confirmed and expanded the discovery, exploring polymorphic phases. These two landmark papers inaugurated the field. Subsequently, the review by Sun et al.^217^ and the study by Wang et al.^264^ consolidated and expanded knowledge, addressing the synthesis and borospherene B_28_. Specialized works, such as Chen et al.^261^ on hydrogen storage and Wu et al.^262^ on superconductivity and growth and characterization studies, demonstrate the diversification of the field. The most recent paper, Chen et al.^263^ on the B_63_ structure, has only 5 citations due to its recent publication but may still have a significant impact. In summary, the stratospheric citations of Mannix et al.^63^ and Feng et al.^195^ confirm the historic nature of the discovery of borophene, while the remaining papers demonstrate its consolidation and ramification into new areas.^201–205^

**Figure 12:**
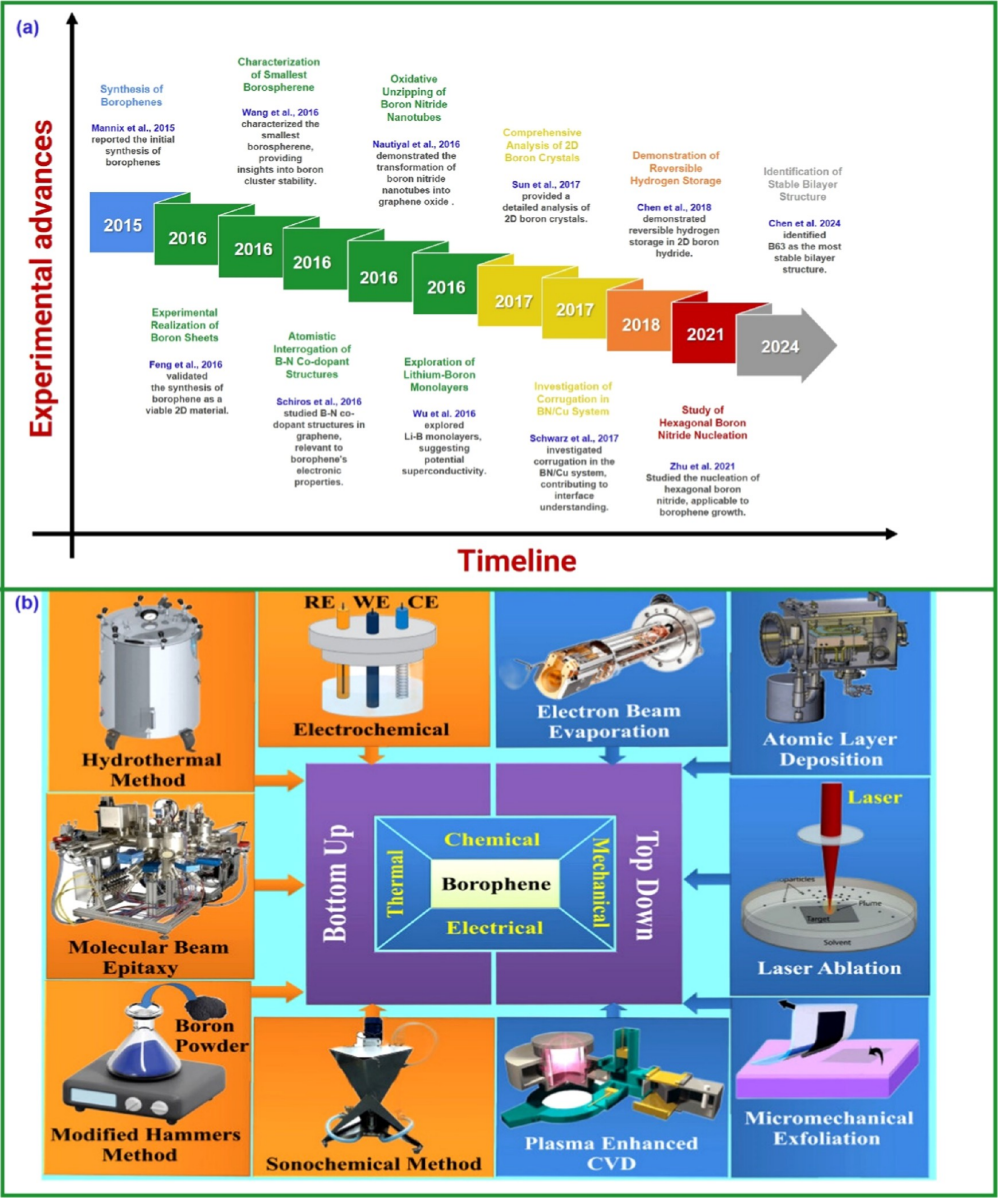
(a) Chronological timeline of experimental advances (2014–2024). (b) Schematic representation of various borophene synthesis techniques. Reproduced with permission from ref 206 Copyright (2025), American Chemical Society.

Michel, Rausch, and Ronsheim^207^ studied the effect of silicon ion implantation on boron diffusion. They showed how manipulating defects (such as point defects and extended defects) influences the diffusion and resistance of the material, something that can be applied to the control of defects in borophene. These effects are important for materials processing and the control of the electronic properties of borophene in nanoelectronic devices. In 1997, Boustani^208^ showed that B_2_–B_14_ clusters form stable hexagonal or pentagonal pyramidal units from *n* ≥ 9, and that quasi-planar multilayer structures can serve as models for nanotubes and planar surfaces, with applications in shielding, neutron absorption and high-temperature semiconductors. Lau and Pandey^58^ expanded knowledge on triangular and hexagonal sheets, confirming metallic or semiconductor stability. Er et al.[Bibr ref27] (2009) showed that doping with alkali metals allows storage of up to 10.7 wt % of H_2_. Tian et al.^209^ and Saxena & Tyson[Bibr ref210] (2010) highlighted the stability of α fullerenes, nanowires, and nanoribbons, while Galeev et al.[Bibr ref211] (2011) and Boustani et al.^112^ elucidated the coexistence of 2D and 3D isomers, essential for the dimensional transition in clusters. Wu et al.[Bibr ref62] (2012) predicted stable and compatible α1 and β1 sheets with MWBNTs, and Liu et al.[Bibr ref60] (2013) proposed synthesis on Au, Ag, MgB_2_, and Cu surfaces, favoring 2D sheets with hexagonal holes. Banerjee et al.^212^ evaluated borophene α1 as an anode for LIBs; Zhou et al.^61^ predicted a new buckled phase with a Dirac cone. Between 2015 and 2017, Wang et al.,^213^ Xu et al.,^214^ Mannix et al.^63^ and Zhang et al.^215^ consolidated the synthesis and experimental characterization of borophene, showing substrate dependence, metallic stability, and anisotropy. Penev et al.^184^ and Li et al.[Bibr ref216] (2016) highlighted superconductivity and dynamic coexistence of 2D/3D isomers; Feng et al.^195^ synthesized stable β_12_ and χ_3_ sheets on Ag(111). Yu et al.[Bibr ref217] (2017) reviewed advances in B_33–_ and B_34–_ clusters, vacancies, and aromaticity. Hou et al.[Bibr ref14] (2020) consolidated controlled synthesis and technological perspectives. Anju and Shiju^218^ and Macilon et al.[Bibr ref219] (2025) analyzed synthesis, electronic, mechanical, and thermal properties, and applications in HER, solar cells, and energy storage, highlighting stability and scalability challenges, consolidating borophene as a promising material for energy and electronic technologies, as shown in Figure 12b. Beyond conventional MBE and CVD, innovative strategies have recently been proposed, including substrate reconstruction-driven growth^220^ and template-assisted ultrathin boron growth,^221^ both of which broaden the accessible polymorph space. Figure 13 shows the CVD synthesis process for the 2D γ-boron films. Figure 13a illustrates the two-zone furnace used to control the temperature and deposit borate and borate oxide onto copper foil.^197^ Figure 13b displays the monolayer structure of γ-Boron, composed of icosahedral B_12_ units and B_2_ dumbbells. Figure 13c shows a continuous monolayer of γ-boron on copper, while Figure 13d shows the polyhedral structure of the γ-boron unit cell. Experimental studies show that borophene’s properties are strongly influenced by its intrinsic structural features formed during epitaxial growth. Vacancy ordering, especially in the β_12_ and χ_3_ phases, not only determines bond connections but also affects charge distribution and conduction routes, leading to anisotropic metallicity.^222^ These vacancies create regions of low atomic density, impacting the elastic modulus and making the material stiff along some directions and more deformable in others. Stresses from substrates like Ag(111) and Au(111) cause local distortions that modify band alignment and phonon dispersion, affecting superconductivity and thermal transport.^223^ Managing these structural features is vital for producing borophenes with consistent properties for technological use.^224^

**Figure 13:**
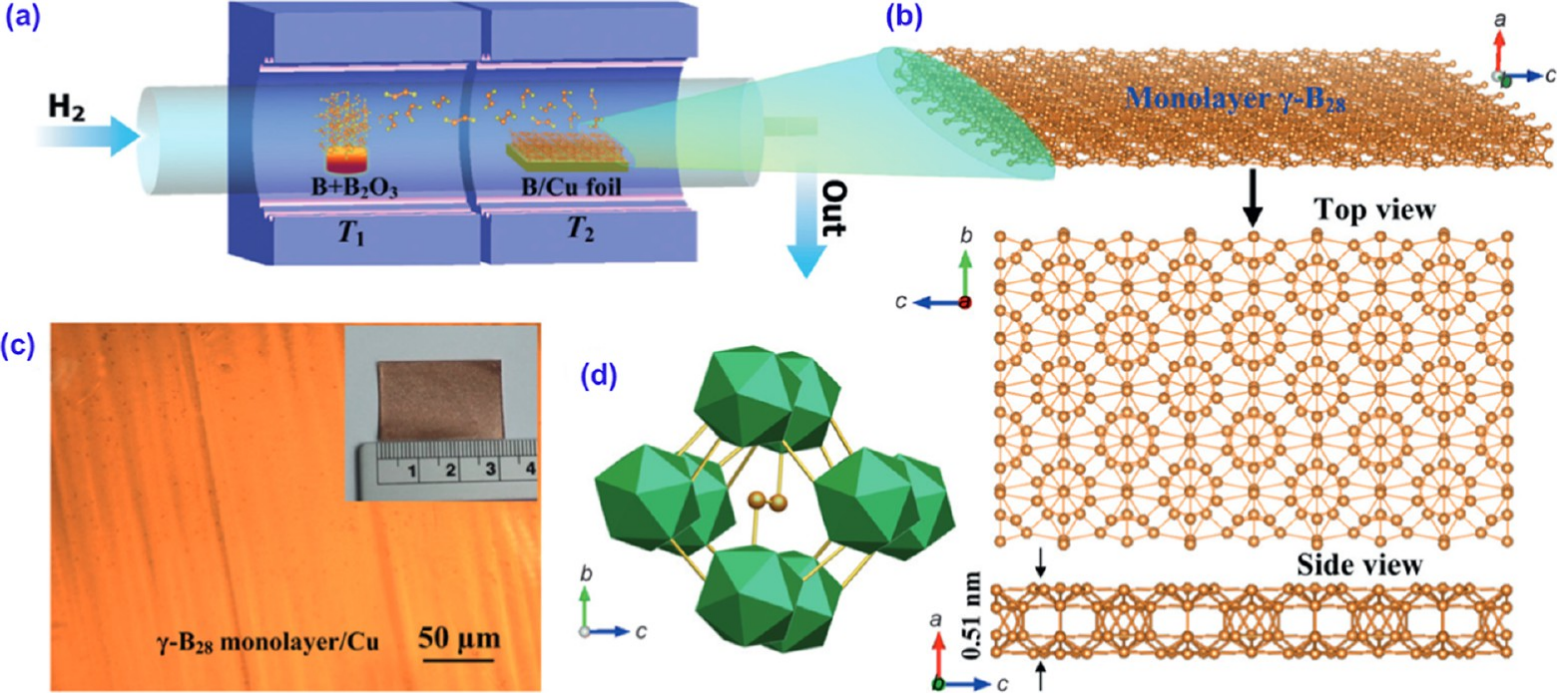
(a–d) Experimental CVD growth of borophene. Reproduced with permission from ref 197 Copyright (2015), Wiley.

## 5 Current Status and Properties

Borophene has rapidly emerged as one of the most technically compelling members of the 2D-materials family, largely due to its structural polymorphism and the strong anisotropy of its physical properties, as shown in Table 4. Unlike graphene, which possesses a strictly hexagonal lattice, and phosphorene, which exhibits a puckered orthorhombic structure, borophene can adopt multiple polymorphs, including β_12_, χ_3_, δ_6_, and vacancy-modulated arrangements, all derived from variations in the density and distribution of hexagonal vacancies.^225^ This polymorphism grants borophene exceptional tunability but also introduces significant challenges related to phase control, reproducibility, and environmental stability.

**Table 4:** Comparing the Unique Properties of Borophene against Graphene, Phosphorene, and Other 2D Materials

property	borophene	graphene	phosphorene	other 2D materials	refs
atomic structure	boron atoms in a honeycomb or other nonhexagonal structures	carbon atoms in a hexagonal lattice	phosphorus atoms in puckered structure	varied (e.g., transition metals, sulfides, etc.)	^12,186,238–243^
electrical conductivity	high, but can vary with structure	exceptional, one of the best conductors	moderate, but anisotropic	varies (e.g., MoS_2_ is semiconducting)	
thermal conductivity	moderate	very high	lower than graphene	varies, generally lower than graphene	
mechanical strength	high, but depends on the structure	extremely strong	moderate, brittle	depends on material (e.g., MoS_2_, weak)	
flexibility	high flexibility and tunability	very flexible	less flexible, brittle	varies (e.g., MoS_2_, flexible)	
band gap	can be tunable, some structures may have a band gap	zero band gap (semimetal)	has a band gap (semiconductor)	varies (e.g., MoS_2_, semiconductor)	
optical properties	can have tunable optical properties	transparent in visible spectrum	strong absorption in the visible range	varies (e.g., MoS_2_, strong light absorption)	
chemical reactivity	highly reactive, especially with oxygen	relatively inert	highly reactive, especially in ambient conditions	varies (e.g., MoS_2_, more reactive)	
synthesis methods	mechanical exfoliation, chemical vapor deposition (CVD), and other methods	CVD, mechanical exfoliation	exfoliation, chemical vapor deposition	chemical vapor deposition, exfoliation, etc.	
applications	sensors, flexible electronics, energy storage	electronics, photonics, energy storage	electronics, photonics, batteries	varied (e.g., MoS_2_ for transistors, catalysts)	

### 5.1 Electronic Behavior and Structure–Property Relationships

Most borophene phases exhibit intrinsic metallicity, distinguishing them from graphene, a zero-bandgap semimetal, and from semiconducting 2D materials, such as phosphorene and TMDC monolayers. Its metallic nature arises from partially filled σ networks combined with delocalized multicenter π systems, both influenced by the electron-deficient character of boron. The electronic properties of borophene are highly sensitive to the structural and external parameters. The density of hexagonal vacancies strongly modifies the fermi surface and transport behavior; for instance, β_12_ displays higher mobility and lower carrier effective mass along vacancy-aligned directions, while χ_3_ tends to be more isotropic but with generally lower mobility.^226^ Defects, such as vacancies, adatoms, and grain boundaries, can reorganize metallic pathways, enhancing catalytic performance but usually reducing conductivity and structural stability. Substrate interactions also play a key role: epitaxial borophene on Ag(111) or Au(111) receives charge transfer that can change carrier concentrations to high, stabilizing certain phases such as β_12_.^227^ On weakly interacting substrates, the intrinsic ordering of phases may change, which complicates reproducibility.

### 5.2 Mechanical, Thermal, and Structural Anisotropy

Borophene exhibits mechanical robustness comparable to or even surpassing that of graphene along specific crystallographic directions but with pronounced anisotropy arising from its vacancy patterns and buckled configurations. The β_12_ phase is relatively flat and presents high stiffness perpendicular to vacancy rows, whereas χ_3_ contains stronger buckling, resulting in higher out-of-plane flexibility but reduced in-plane stiffness. Strain engineering has a particularly strong impact: uniaxial strain of only a few percent can induce Dirac-like dispersions or significantly modify Fermi velocity, in some cases with much higher sensitivity than observed in graphene.^228^ This differentiates it from graphene, a zero-bandgap semiconductor with extremely high mobilities, and phosphorene, which has a direct bandgap tunable between 0.3 and 2.0 eV, useful for transistors.^229^ Thermal conductivity predictions for borophene vary widely. Although theory suggests potentially very high thermal transport, experimental measurements remain lower and highly phase-dependent, limited by phonon scattering at vacancy lines and grain boundaries. This behavior contrasts with TMDCs, where heavy atom masses dominate phonon dispersion, and with graphene, whose thermal conductivity is defined by long-wavelength phonons and minimal scattering. TMDCs, such as MoS_2_, have stable direct bandgaps (∼1.2–1.9 eV) and are already integrated into optoelectronics.^230^ Mechanically, borophene combines high strength with flexibility, albeit anisotropically, while graphene maintains its position as the most robust material known. Regarding thermal conductivity, graphene remains the benchmark (2000–5000 W/m·K), while borophene exhibits inconsistent values: theoretical predictions suggest exceptional performance, but experimental measurements reveal strong dependence on phase and crystalline quality.^231^

### 5.3 Chemical Reactivity, Functionalization, and Stability

Borophene is highly chemically reactive, undergoing rapid oxidation in air, often within seconds or minutes of exposure, requiring strategies such as functionalization (borophane and fluorination) or encapsulation (h-BN and graphene) for preservation.^232^ The β_12_ phase demonstrates slightly better oxidation resistance compared to χ_3_, primarily due to its flatter geometry and reduced density of reactive sites. Chemical functionalization—through hydrogenation, fluorination, or formation of borophane—can stabilize borophene by saturating out-of-plane bonds, altering the electronic structure, and improving environmental durability. In borophane, partial rehybridization from sp^2^-like to sp^3^-like bonding reduces metallicity but significantly enhances resistance to oxidation.^233,234^ Whereas phosphorene degrades mainly via photo-oxidation, borophene’s degradation is dominated by chemical oxidation and hydrolysis. TMDCs, with fully saturated chalcogen layers, are comparatively inert, highlighting the need for encapsulation or functionalization strategies to ensure borophene stability.

### 5.4 Position Within the 2D Materials Landscape

Borophene occupies a distinctive position within the broader 2D-materials ecosystem. Compared to graphene, borophene offers direction-dependent metallicity and high structural tunability but lacks the environmental stability and scalable synthesis pathways that have propelled graphene-based technologies. Relative to phosphorene, borophene exhibits superior mechanical strength and metallic conductivity, although both materials face similar instability challenges under ambient conditions. When contrasted with TMDCs, borophene’s metallic character and strong electron–phonon coupling make it more suitable for applications requiring high conductivity, plasmonic behavior, or catalytic activity, while TMDCs remain preferred for semiconducting and optoelectronic applications.^235^ Borophene’s combination of low mass density, mechanical anisotropy, robust electronic conduction, and defect-tolerant catalytic performance positions it as a promising candidate for hydrogen evolution catalysis, ultrathin metallic interconnects, high-frequency plasmonics, and structural reinforcement in advanced composites.^236^ Current knowledge of borophene shows that its properties are driven by structural motifs, not just elemental makeup. The amount and arrangement of vacancies influence the electronic topology and support highly directional metal bands, crucial for ultrathin interconnects. Structural anisotropy also causes different mechanical behaviors, with elastic moduli varying significantly between directions, and impacts heat conduction pathways, affecting heat dissipation and conversion.^237^ Residual substrate strain can alter electron–phonon interactions, stabilizing phases that are otherwise metastable. Therefore, borophene’s performance depends on precise structural engineering, highlighting the importance of controlled synthesis, stress management, and defect control for scalable, robust applications.

## 6 Applications and Commercialization of Borophene

Borophene combines exceptional electrical conductivity, lightweightness, and high specific strength, making it promising in several technology sectors. Recent experimental advancements support borophene’s potential in energy storage, particularly in supercapacitors, Li–S batteries, and anodes.^244–246^ These experimental results highlight borophene’s unique properties, such as high conductivity and large surface area, which make it ideal for next-generation energy storage systems. In electronics, it could enable high-performance transistors and flexible devices in a market estimated to be worth US$40 billion by 2025 from IDTechEx (2023) and Markets & Markets (2024). Its potential for room-temperature superconductivity could also impact energy transmission and storage systems, integrating it into the renewable energy market, projected to be worth US$1.5 trillion (Figure 14a). In the aerospace and automotive sectors, borophene-based composites could create lighter and more efficient structures, tapping into the global composite materials market, valued at US$90 billion in 2020 and expected to grow 7.5% annually through 2027 (Figure 14a). In the biomedical sector, its surface properties favor advances in biosensors and drug delivery systems, markets estimated at US$36 billion and US$2.2 trillion, respectively, with applications in health monitoring, motion detection, and human–machine interfaces (Figure 14c). In water treatment, it stands out for its efficiency in desalination, in a market worth US$30 billion in 2020, with a CAGR of 6% until 2025, as well as its potential in sensors and energy storage (Figure 14b). Despite its high potential, borophene synthesis remains limited to micrometric samples via molecular beam epitaxy or chemical vapor deposition, hindering large-scale production (Figure 14a). Once issues of scalability, cost, and environmental impact are overcome, borophene could establish itself as a key material in disruptive technologies with significant multisectoral economic impact in the next decade. Life cycle assessments (LCAs) will be crucial to guide sustainable production, ensuring that technological innovation goes hand in hand with the minimization of environmental impacts. Furthermore, its versatility will allow the integration of electronic, energy, and mechanical functionalities into flexible and compact platforms, from wearable devices and portable electronics to advanced energy storage and conversion systems. The combination of borophene’s unique properties with advanced 2D synthesis and integration techniques could catalyze new device architectures, fostering innovation in sectors such as healthcare, mobility, robotics, and renewable energy, solidifying it as a pillar of the next generation of technology.

**Figure 14:**
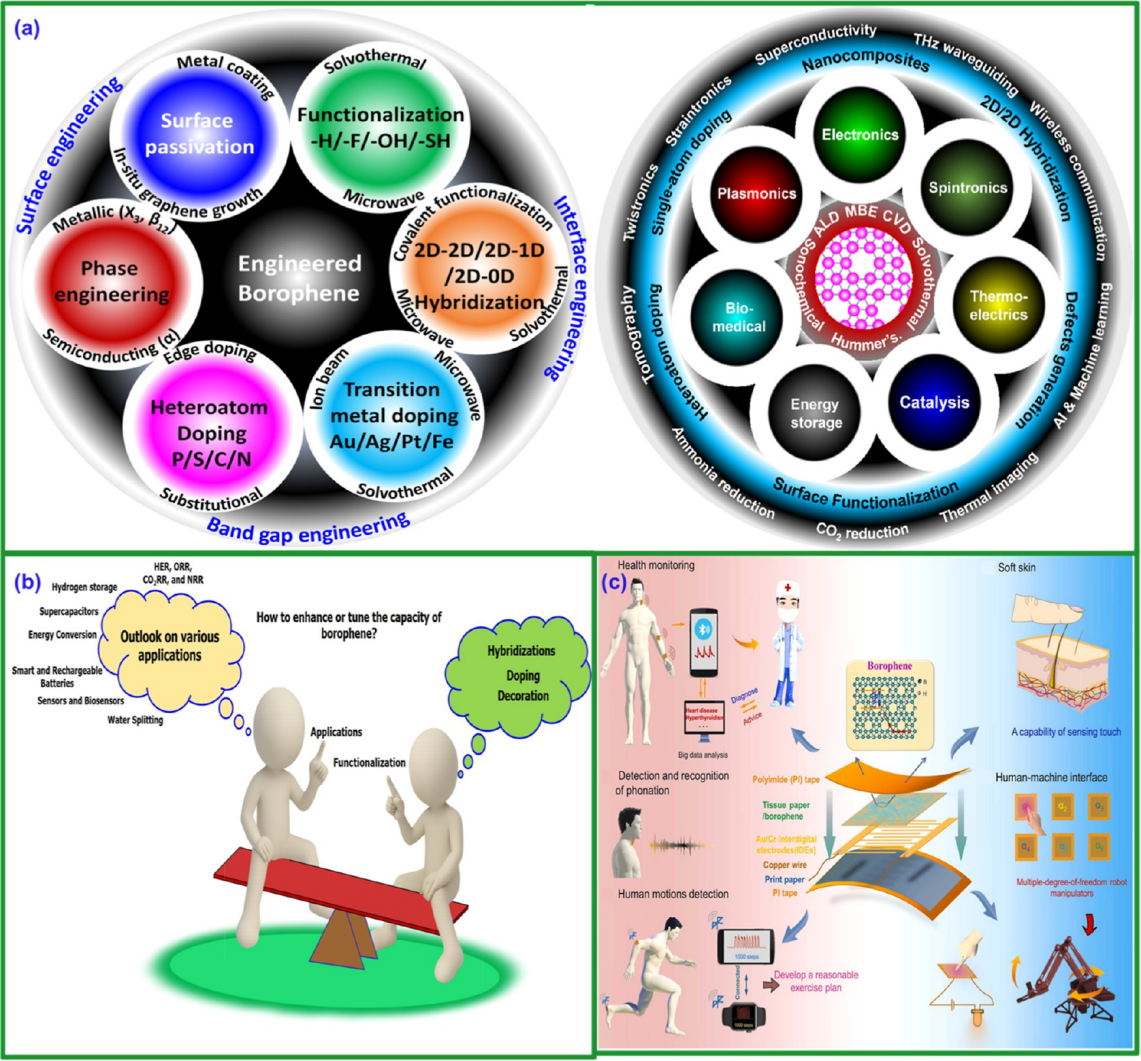
(a) Strategies for the development of engineered borophene and its applications. Reproduced with permission from ref 247 Copyright (2025-License: CC BY), Wiley. (b) Potential of borophene for next-generation applications. Reproduced with permission from ref 10 Copyright (2025), Royal Society of Chemistry. (c) Diverse applications of borophene in pressure sensors Reproduced with permission from ref 248 Copyright (2022), Elsevier.

### 6.1 Market Potential

The 2D materials era is experiencing strong expansion, with a market projected to reach US$3.59 billion by 2030 from 360iResearch (2024),^249^ as shown in Figure 15a. Graphene is leading this movement, jumping from US$0.29 billion (2024) to US$2.34 billion (2030) from BCC Research (2024) and Value Market Research (2024),^250,251^ as shown in Figure 15b. Borophene, although still primarily used in research, is already commercialized by SUNUM and PowderNano at prices between US$440,000 and US$690,000 per gram.^252–254^ Production is limited but could grow rapidly as commercial applications are discovered. By 2030, the material is estimated to capture up to 15% of the 2D materials market, generating approximately US$500 million annually, driven by sectors such as flexible electronics, energy storage, and advanced coatings.^253,255^ Products already available include NanoPro’s Borophene automotive coatings, with 40% greater gloss than graphene, extreme hydrophobicity (180°), and heat resistance up to 3000 °C. Globally, universities, institutes, and companies are seeking to overcome the challenges of synthesis and scalability. Northwestern University leads silver synthesis, while the Chinese Academy of Sciences is developing production via liquid exfoliation for energy and catalysis. BASF SE is investing in composites and advanced batteries; Panjab University is exploring environmental applications in membranes and photocatalysis; and Tsinghua University is focusing on electronics, optoelectronics, and quantum computing. Government support is also crucial for development in 2D materials.^254^ The main obstacles are sensitivity to oxidation and the complexity of CVD synthesis, which requires extreme vacuum and temperature conditions. However, borophene, abundant and potentially sustainable, could revolutionize areas such as ultrasensitive biomedical sensors^256–258^ and optoelectronic devices.^10,259,260^ As researcher Emily Tanaka (MIT) summarizes: “Borophene is not just another 2D material, it is a technology platform with the potential to redefine multiple industrial sectors.” With increasing investment and advances in stabilization, the next five years will be decisive for its definitive transition from the laboratory to market.

**Figure 15:**
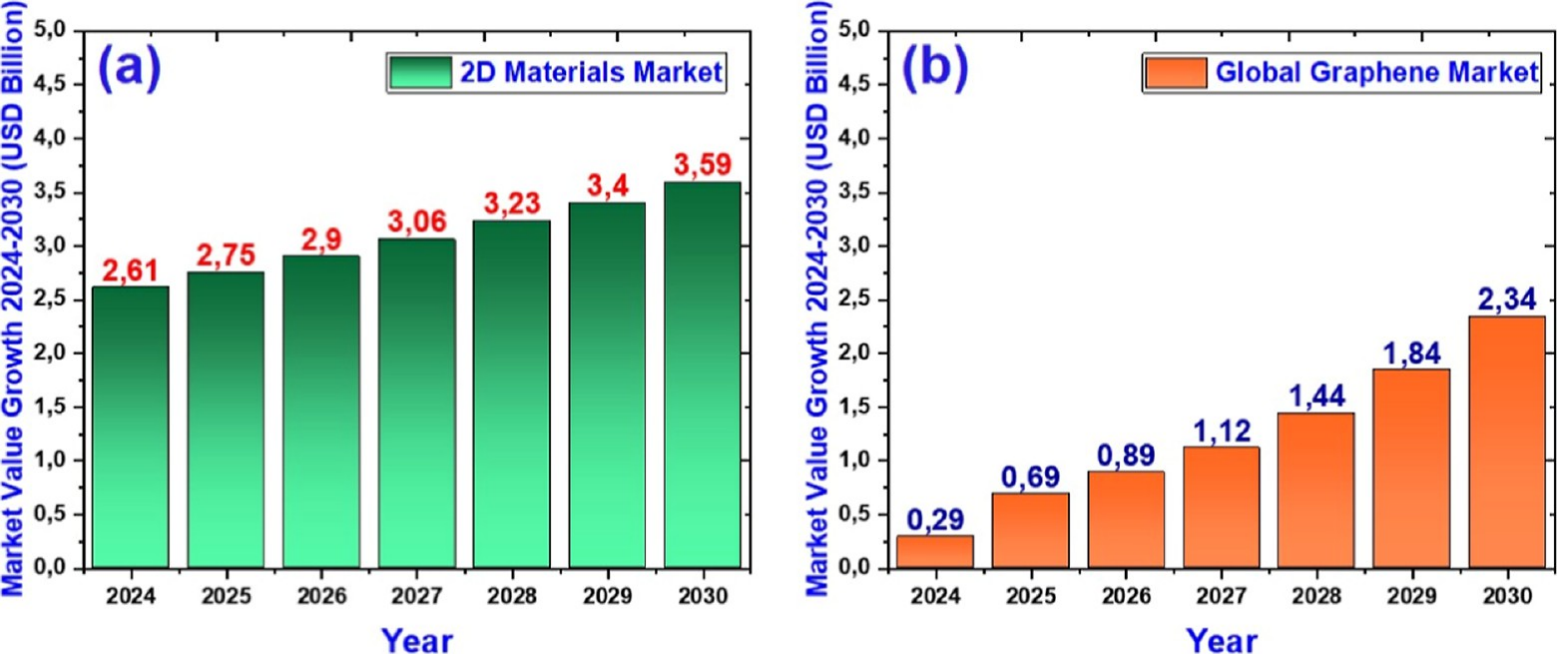
From predictions to (a) 2D materials market and (b) global graphene market: the rising age of 2D materials.^249–251^

## 7 Limitations and Outlook

Borophene’s rapid ascent, from theoretical curiosity to a growing platform for next-generation technologies, has been driven by unprecedented advances in modeling, synthesis, and characterization, as shown in Figure 16a. Yet its transition from laboratory discovery to industrial relevance remains hindered by several fundamental challenges. The first and most critical issue is environmental instability. Freestanding borophene rapidly oxidizes and reconstructs upon exposure to ambient conditions, necessitating stabilization via encapsulation (e.g., h-BN and graphene), chemical functionalization (borophane and halogenation), or substrate-induced passivation. Developing scalable and durable protection strategies remains essential for any practical deployment. A second major challenge lies in phase control during the synthesis. Borophene’s polymorphism, one of its greatest advantages for property engineering, complicates precision growth. Current MBE-based routes suffer from sensitivity to temperature, flux, and substrate crystallography, often yielding mixed-phase or substrate-dependent structures. To unlock predictable and tunable properties, researchers must develop synthesis frameworks capable of deterministic polymorph selection, including solution-phase precursors, CVD pathways, and substrate engineering approaches that control surface energy, lattice matching, and electron donation. A related constraint is scalability. Nearly all reported borophene synthesis occurs under ultrahigh-vacuum (UHV) conditions on metallic substrates, producing only micrometer-scale samples. Industrial translation demands new manufacturing paradigms, such as atmospheric-pressure CVD, catalytic template transfer, and roll-to-roll growth on flexible supports. Achieving continuous, large-area production while preserving phase purity and preventing oxidation is arguably the central bottleneck in borophene’s commercialization. Another frontier involves substrate and heterostructure engineering. Because freestanding borophene is difficult to stabilize, the substrate is not merely a support surface; it is often an active electronic, structural, and thermodynamic determinant. Designing patterned, dielectric, catalytic, or strain-graded substrates could enable unprecedented control over phase emergence, doping, strain engineering, and device integration. Layer-by-layer stacking with other 2D materials, graphene, MoS_2_, and h-BN, may further provide routes to hybrid architectures with tailored bandgaps, enhanced chemical resistance, or improved charge transport. Finally, deeper synergy among computational modeling, machine learning, and experiment will be essential. The synergy between theory and experimentation is essential. Advanced platforms such as The Mat3ra Platform, VASP, Quantum ESPRESSO, ABINIT, and machine learning approaches have enabled the prediction of new polymorphs, property optimization, and experimental guidance. High-throughput DFT screening, reinforcement-learning-based synthesis optimization, and AI-accelerated discovery of new borophene polymorphs can guide experimentalists toward viable structures before growth begins. Likewise, in situ spectroscopy, automated MBE/CVD control, and physics-informed neural networks enable real-time feedback during synthesis. This computational–experimental loop will reduce costs, accelerate materials discovery, and bring industrial fabrication within reach. Substrate engineering and 2D heterostructures also offer avenues for stabilization and property modulation. In the coming years, the focus should be on integrating borophene into practical devices through scalable synthesis and controlled functionalization. Looking forward, a cohesive roadmap is emerging: (1) atomically precise phase control, (2) robust stabilization under ambient conditions, (3) tunable substrate engineering for property modulation, (4) integration into functional devices, (5) scalable synthesis through CVD and roll-to-roll manufacturing, and (6) a unified computational–experimental ecosystem for accelerated design, as shown in Figure 16b. As these elements converge, borophene is poised to evolve from a scientific achievement into a manufacturable, multifunctional material platform capable of transforming electronics, catalysis, energy storage, sensing, and composite engineering. Machine learning is used for polymorph prediction, high-throughput DFT screening, autonomous synthesis platforms, and closed-loop optimization. Its ultimate success will rely on harmonizing fundamental understanding with technological scalability, ensuring that the exceptional properties predicted and observed at the nanoscale can be delivered reliably on the macroscale.

**Figure 16:**
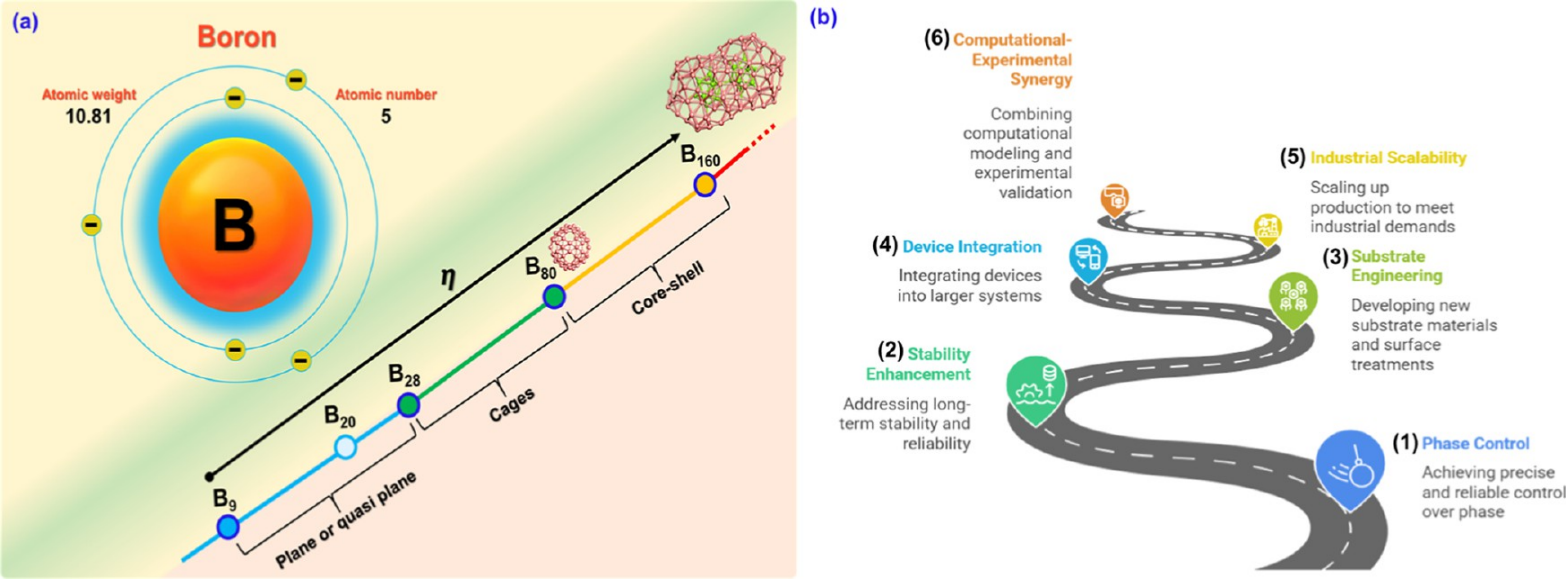
(a) Progress over the past three decades and (b) roadmap pillars for borophene research and technological translation.

## 8 Conclusions

Borophene has rapidly evolved from a theoretically proposed structure to a validated 2D material with unique electronic, mechanical, and chemical characteristics. The convergence of advanced computational modeling with surface-assisted synthesis, particularly on Ag(111), has been fundamental for establishing its structural diversity and elucidating its anisotropic and phase-dependent properties. These developments underscore the central role of theory–experiment integration in guiding the discovery and rational design of boron-based 2D systems. Despite these advances, significant barriers still limit the translation of borophene into practical technologies. Large-area synthesis remains challenging due to the strict requirements of UHV-based growth and the difficulty of isolating single-phase domains with high structural uniformity. Environmental instability—marked by rapid oxidation and lattice reconstruction under ambient conditions—further constrains device integration, highlighting the need for robust stabilization strategies such as encapsulation, controlled functionalization, and substrate-mediated passivation. Progress in polymorph control, including deterministic phase selection through engineered substrates and scalable CVD-based routes, will be critical for expanding its technological relevance. Even with these limitations, borophene already demonstrates compelling potential across emerging application spaces. Heterostructures incorporating borophene have shown enhanced sensitivity and operational stability in biosensing platforms. In energy-storage systems, advances in defect engineering, doping, and layered architectures have begun to address the intrinsic challenges in capacity retention and electrochemical durability. Catalytic studies further reveal promising activity in both electrocatalysis and photocatalysis, particularly when borophene is integrated with transition-metal centers or employed in hybrid configurations. Overall, the field is approaching a pivotal stage. Overcoming bottlenecks associated with stability, phase control, and scalable manufacturing will be essential for enabling real-world applications. Given its combination of structural tunability, high carrier mobility, chemical reactivity, and compatibility with heterostructure engineering, borophene is poised to become a versatile platform for next-generation technologies in sensing, catalysis, flexible electronics, and energy conversion. Continued progress in synthesis, theoretical modeling, and interface engineering is expected to accelerate its transition from an emerging scientific discovery to an applied material with a broad technological impact.

## Supplementary Material



## Notes

Title, authors, year, affiliations, country, journal, keywords, and citations (CSV)
